# Supplementary material for: Orientation of the N- and C-Terminal Lobes of the Myosin Regulatory Light Chain in Cardiac Muscle
Source: Biophys J. 2015 Jan 20;108(2):304–14. doi: 10.1016/j.bpj.2014.11.049 (PMC4302210; doi:10.1016/j.bpj.2014.11.049)
Supplement: Document S1. Supporting Materials and Methods, seven figures, and three tables [file mmc1.pdf]

Biophysical Journal

Supporting Material

# Orientation of the N- and C-Terminal Lobes of the Myosin Regulatory Light Chain in Cardiac Muscle

**Thomas Kampourakis, Yin-Biao Sun, and Malcolm Irving**

## Supplemental Text

### Dependence of RLC orientation distributions on the choice of RLC reference structure

The *in situ* orientation distributions of the N- and C-lobes of the RLC shown in **Figs. 3** and **4** of the main text were calculated using maximum entropy (ME) analysis from the measured order parameters (**Table S2**) and the RLC structure of scallop striated muscle myosin with bound ADP (1SR6(1)). These ME calculations were repeated using the five other reference structures in **Fig. S3**. For the order parameters recorded for the N-lobe probes in relaxation and active isometric contraction, this procedure was successful for five of the six reference structures. For the remaining structure, from scallop smooth muscle myosin (3PN7(mol2) (2)), which has an internal bend in the D helix and a distinct $\beta_{DB}$ value (**Fig. S3B**), no distribution of N-lobe orientations could reproduce the measured order parameters, suggesting that the *in situ* structure of cRLC in heart muscle in these conditions is different from that in the 3PN7(mol2) structure. In rigor conditions, three of the six crystallographic structures were inconsistent with the measured order parameters for the N-lobe probes, 3PN7(mol2), chicken skeletal myosin (2MYS (3)), and a scallop striated muscle myosin with bound ADP.$VO_4$ (1QVI (4)).

N-lobe orientation distributions calculated using the two reference structures in addition to 1SR6 that fitted the measured order parameters from the N-lobe probes in all conditions (**Fig. S4**) showed the same three peaks as the 1SR6-based analysis (**Fig. 3**), with somewhat different peak positions and relative amplitudes, as expected from the slightly different folds of the N-lobe (**Figs S3A** and **S3B**). The mean and SD of the peak $(\beta,\gamma)_{DB}$ positions of the N-lobe in relaxing conditions calculated from the five structures that fitted the measured order parameters were (50° ± 10° , -60° ± 30°), (75° ± 20° , 50° ± 20°) and (130° ± 30°, -40° ± 10°). Thus peak N3 is more variable in β, reflecting its tendency to partially merge with peak N1 for some *in vitro* structures. Peak N1, conversely, is more variable in γ, reflecting a tendency to merge with the (180°-β, 180°+γ) dipole-related equivalent of N2.

For the C-lobe probes, the maximum entropy analysis was successful for all six reference structures. In general the resulting orientation distributions were similar for the six structures, with mean ± SD $(\beta,\gamma)_{EG}$ values of (30° ± 5°, 20° ± 20°), (50° ± 10°, -60° ± 5°), (90° ± 5°, 35° ± 5°) and (125° ± 5°, -30° ± 5°) for peaks C1-C4. The smaller SDs compared with those reported above for the N-lobe probes reflect the fact that the structure of the C-lobe is more highly conserved than that of the N-lobe (**Fig. S3**). Example orientation distributions for relaxation, active isometric contraction, and rigor calculated using the 3PN7 and 2BL0 reference structures are shown in **Fig. S4**.

### The effects of experimental errors in the measured order parameters on the 2D ME distributions

The variability in the ME maps was simulated by Monte Carlo analysis. The error distribution of each measured order parameter was modeled as a normal distribution using its experimental mean and SD. The resulting eight normal distributions for each experimental condition were randomly sampled to give a new set of eight $\langle P_2 \rangle$ and $\langle P_4 \rangle$ values that differ from the experimental means used for the original 2D ME calculations in a way that accurately reproduces the experimental variability in the $\langle P_2 \rangle$ and $\langle P_4 \rangle$ values in that condition. Ten example results of the application of this procedure are shown in Fig. S6 for the N lobe, and in Fig. S7 for the C lobe. These ten 2D ME distributions are qualitatively similar in each case, but in general there is greater variability in the amplitude and width of each peak than in the peak position. The panel at the lower right of Figs S6 and S7 shows the

SD of the ten distributions. To a first approximation these SD plots are scaled down versions of the original 2D ME maps shown in Figs 3 and 4 of the main paper, which were calculated from the mean value of each order parameter. Thus the errors in the 2D ME maps arising from the experimental variability of the measured order parameters are sufficiently small that they do not affect the conclusions of the present paper.

## Supplemental Figures

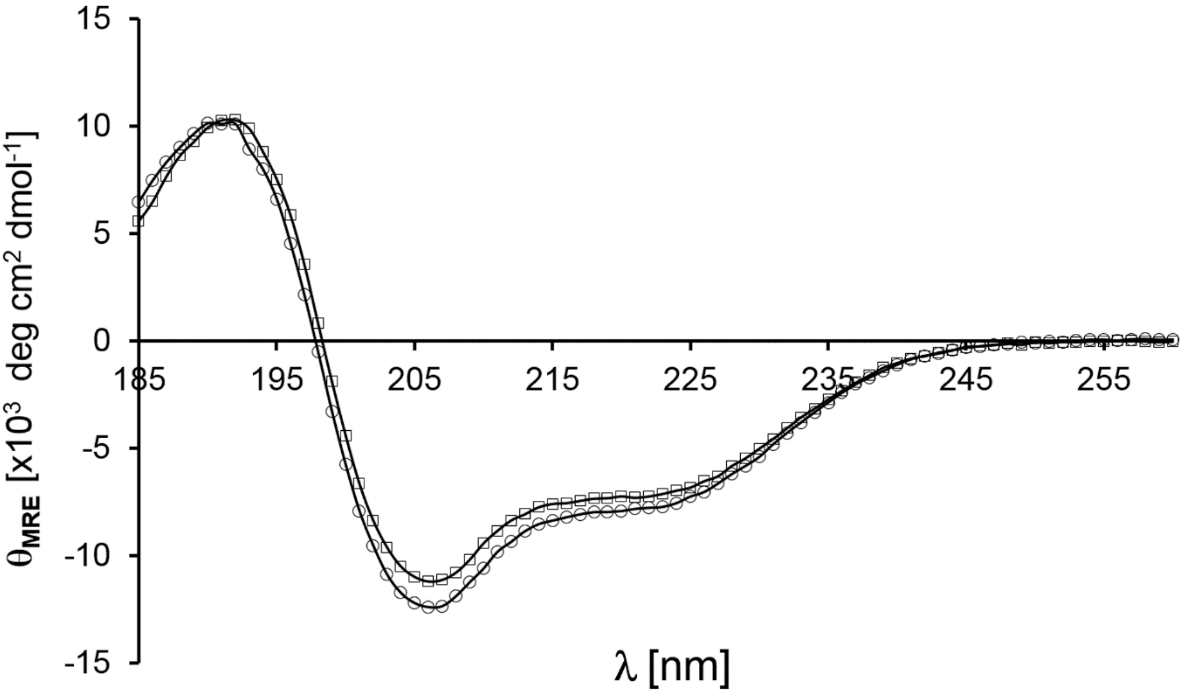


**Figure S1. CD spectra of wild-type and BSR-labeled RLC.**

Far-UV circular dichroism (CD) spectra of wild-type cRLC (squares with interpolated lines) and BSR-cRLC-G (circles with interpolated lines). Both spectra are typical for α-helical proteins with a high content of unfolded regions. The α-helical content in the BSR-cRLC-G is increased by 2% compared to wild-type, calculated using the standard equation for $[\theta]_{MRE}$ at 222 nm.

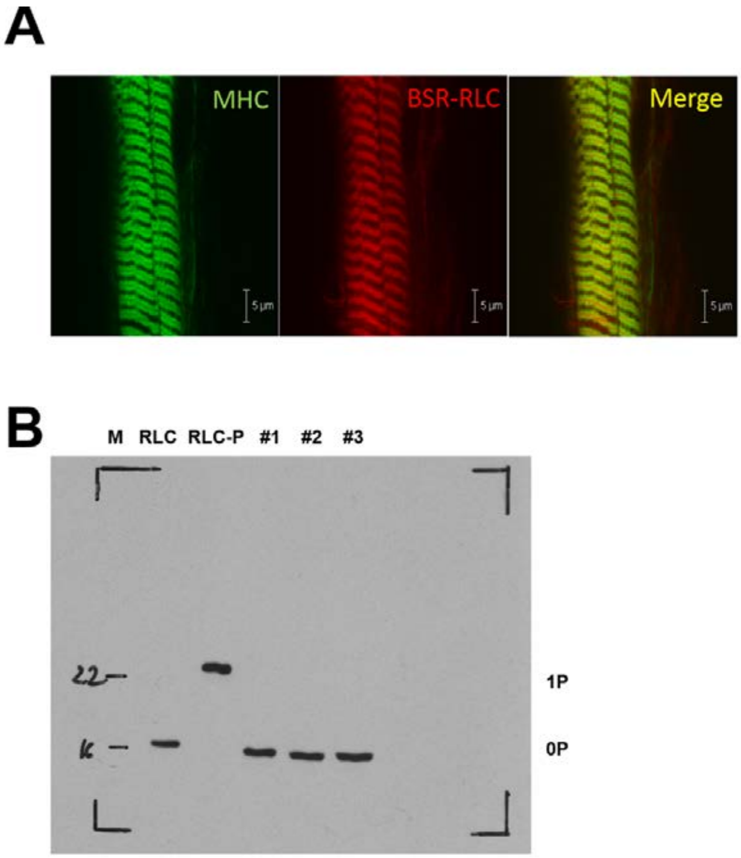


**Figure S2. Sarcomeric localisation of BSR-cRLC and phosphorylation level of endogenous rat cRLC.**

(**A**) Confocal microscopy image of a rat right ventricular trabecula after partial replacement of endogenous cRLC with BSR-cRLC-G (red channel) counterstained against myosin heavy chain (green). (**B**) The phosphorylation level of endogenous cRLCs from three different skinned rat trabecula preparations, determined by Phos-tag$^{TM}$ (5) SDS-PAGE followed by Western blot against cRLC (*RLC:* unphosphorylated recombinant cRLC; RLC-P: mono-phosphorylated recombinant cRLC; *#1=#3*: skinned trabeculae samples from three different hearts).

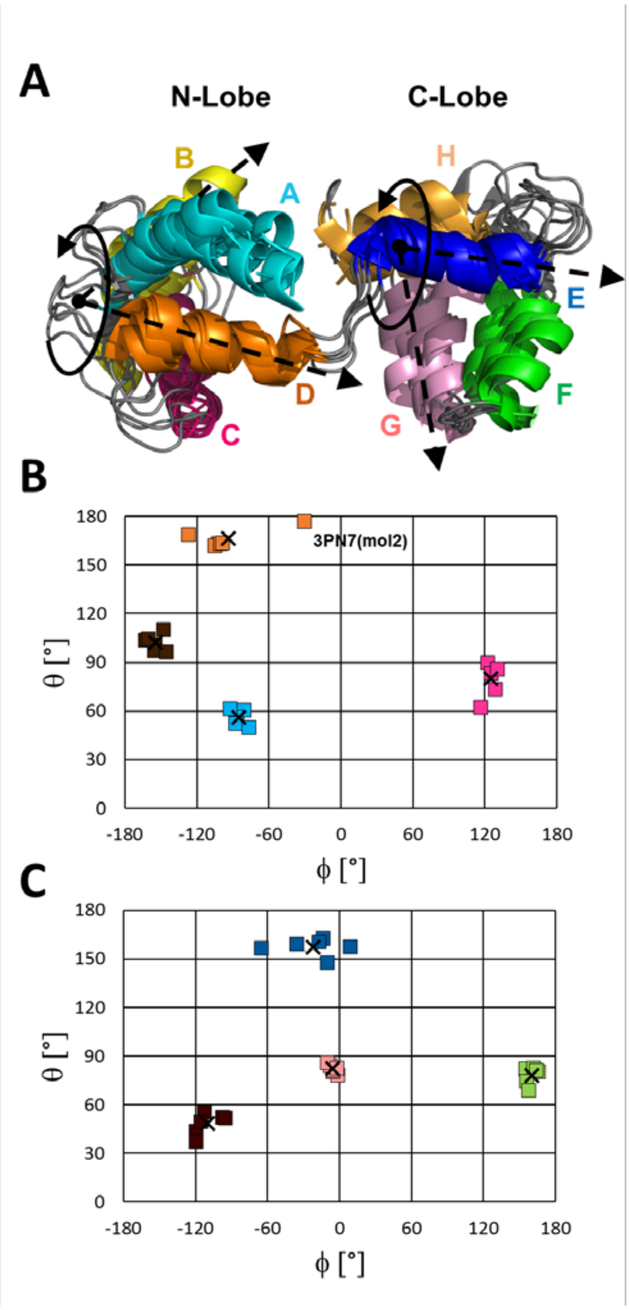


**Figure S3. BSR dipole orientations in six reference structures of the RLC.**

Dipole orientations (θ,ϕ) for BSR probes on the RLC N- and C-lobes calculated from different crystal structures (PDB entries 2MYS (3), 1SR6 (1), 3PN7 (2), 1QVI (6), 2BL0 (7)). (**A**) Structural alignment of RLC crystal structures: A-helix: cyan; B-helix: yellow; C-helix: magenta; D-helix: orange; E-helix: blue; F-helix: green; G-helix: pink; H-helix: light orange. The references axes describing the molecular references frames for the N- and C-lobe (DB- and EG-helix frame) are indicated by dashed arrows. (**B**) Probe dipole orientations on the N-lobe from different crystal structures with same colour coding as in (**A**); probe dipole of BSR crosslinking helices B and C shown in brown. (**C**) Probe dipole orientations on the C-lobe (FG-helix: brown). Black crosses indicate average probe dipole orientations for all structures.

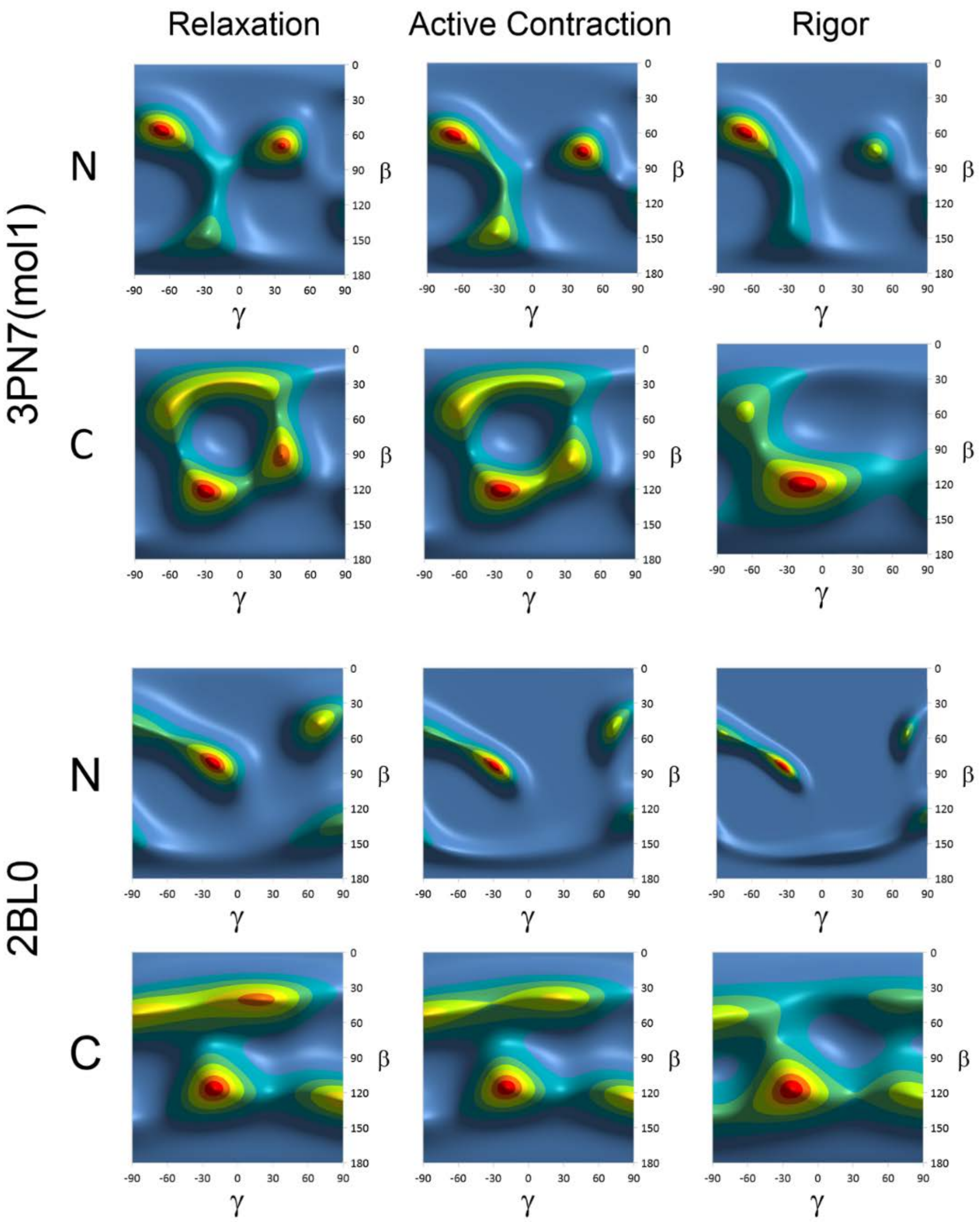


**Figure S4. Maximum entropy distributions calculated using reference structures 3PN7(mol1) and 2BL0.**

Maximum entropy distributions were calculated from order parameters for the N- and C-lobe RLC probes during relaxation, active isometric contraction and rigor (**Table S2**) using reference structures 3PN7(mol1) and 2BL0.

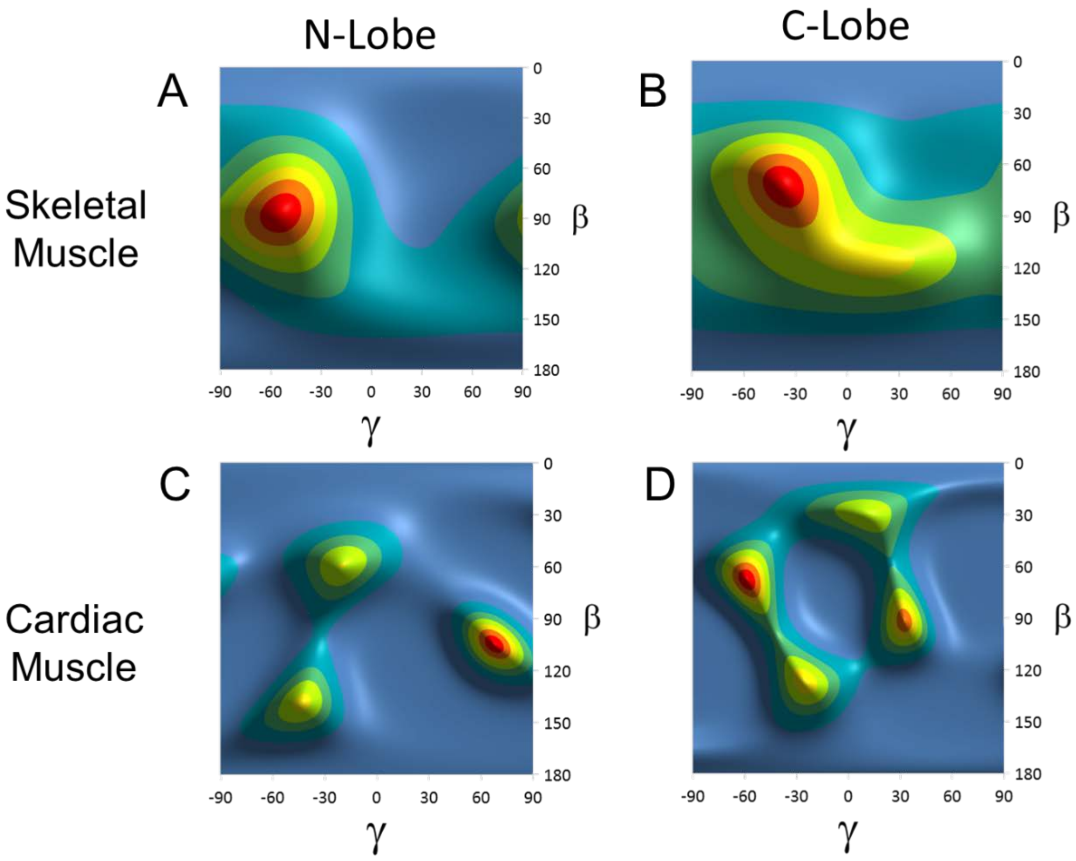


**Figure S5. Comparison of orientation distributions of the N- and C-lobes of the RLC in relaxed skeletal and cardiac muscle.**

(**A** and **B**). Maximum orientation distributions of the N- and C-lobes respectively of the RLC region of the myosin heads in relaxed skeletal muscle from the data of Romano et el ((8)) and Brack et al ((9)) transformed into the DB and EG coordinate frames used in the present work. (**C** and **D**). Maximum orientation distributions of the N- and C-lobes respectively of the RLC in relaxed cardiac muscle from Figs. 3 and 4 of the main text.

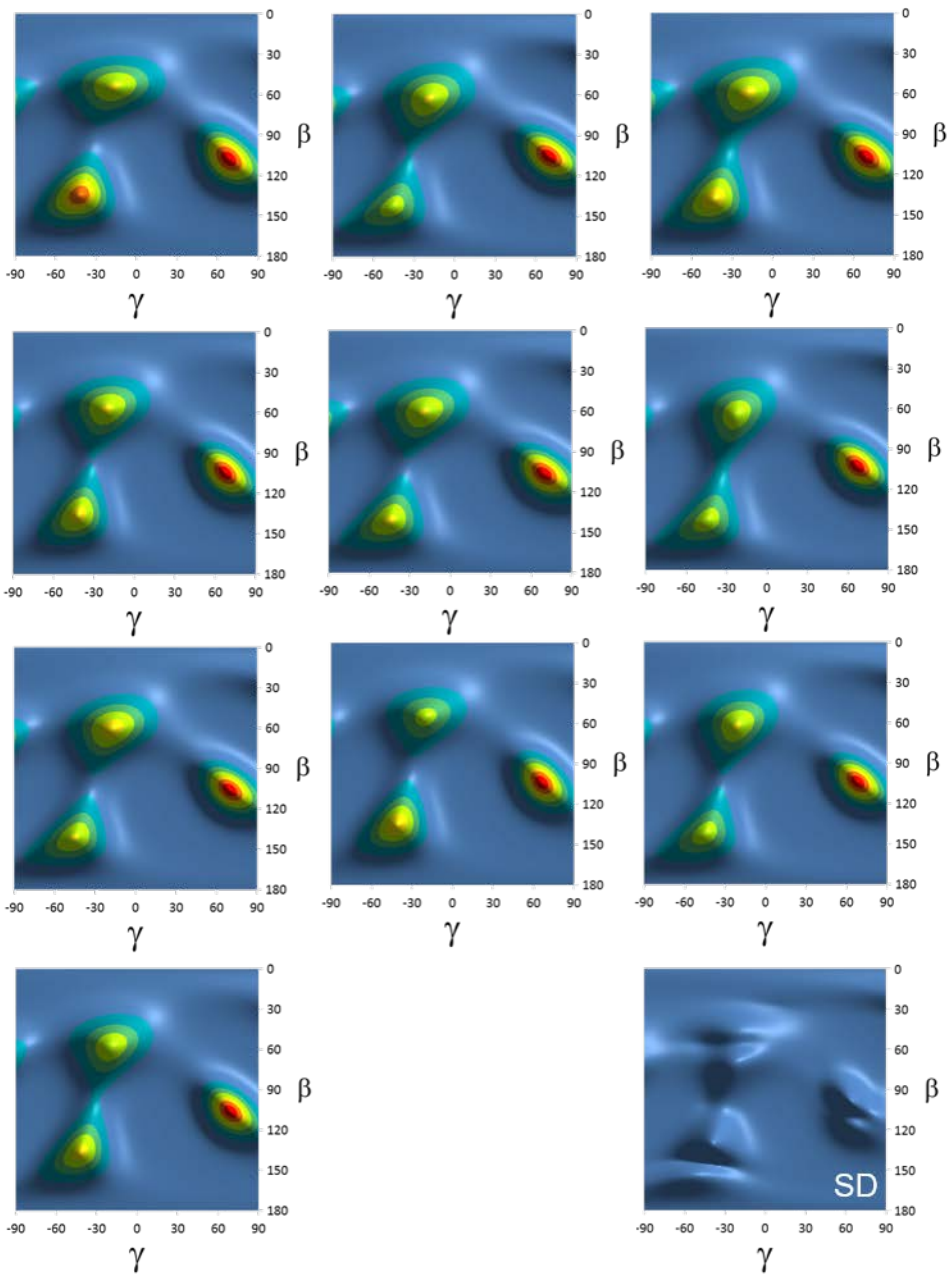


**Figure S6. Reproducibility of maximum entropy orientation distributions of the RLC N-lobe ($(\beta,\gamma)_{DB}$) in relaxation, calculated using RLC coordinates from PDB entry 1SR6.**

In order to assess the effects of measurement errors in the order parameters in **Table S2**, the mean values used for Fig. 3 in the main text were replaced by values chosen at random from normal distributions with the same mean and standard deviation. This procedure was repeated to give the ten contour maps shown to the top and left; the panel at the lower right, labeled SD, shows the standard deviation of these ten distributions.

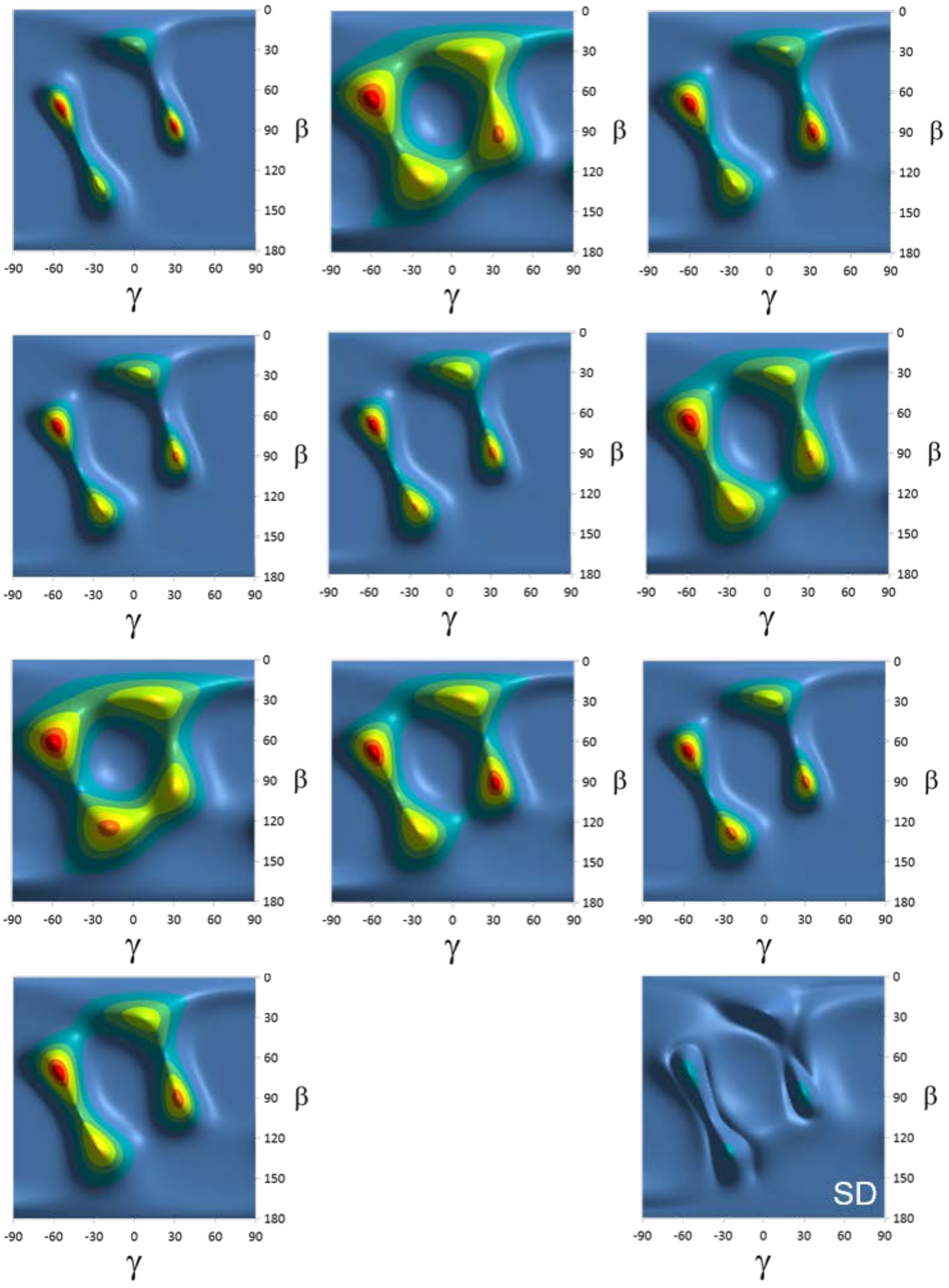


**Figure S7. Reproducibility of maximum entropy orientation distributions of the RLC C-lobe (($\beta,\gamma)_{EG}$) in relaxation, calculated using RLC coordinates from PDB entry 1SR6.**

In order to assess the effects of measurement errors in the order parameters in **Table S2**, the mean values used for Fig. 4 in the main text were replaced by values chosen at random from normal distributions with the same mean and standard deviation. This procedure was repeated to give the ten contour maps shown to the top and left; the panel at the lower right, labeled SD, shows the standard deviation of these ten distributions.

**Supplemental Tables**

**Table S1. Direct Comparison between EDTA- and CDTA exchange of BSR-cRLC-BC**

| | | **CDTA Exchange** | **EDTA Exchange** |
|---|---|---|---|
| | Exchange Rate [%] | 50 ± 5 | 12 ± 2 |
| REL | $<P_{2d}>$ | 0.8063 ± 0.0143 | 0.8079 ± 0.0162 |
| | $<P_2>$ | -0.0009 ± 0.0078 | -0.0106 ± 0.0067 |
| | $<P_4>$ | -0.0237 ± 0.0130 | -0.0210 ± 0.0136 |
| ACT | $<P_{2d}>$ | 0.8015 ± 0.0151 | 0.8027 ± 0.0110 |
| | $<P_2>$ | 0.0853 ± 0.0050 | 0.0762 ± 0.0043 |
| | $<P_4>$ | -0.0212 ± 0.0150 | -0.0175 ± 0.0085 |
| | Force Before Exchange [kPa] | 36.5 ± 3.5 | 37.6 ± 2.2 |
| | Force After Exchange [kPa] | 31.4 ± 2.7 | 33.6 ± 2.5 |
| | Force Recovery [%] | 87 ± 6 (n=7) | 89 ± 3 (n=5) |

Statistical significance of differences between values was assessed using the paired Student's t-test: †$P < 0.05$; ‡$P < 0.01$; *$P < 0.001$

**Table S2. Order parameters for the N- and C-lobe RLC probes during relaxation, active isometric contraction and rigor.**

Order parameters $<P_{2d}>$, $<P_2>$ and $<P_4>$ for the orientation of the BSR-cRLC probes during relaxation, active isometric contraction and rigor in permeabilized right ventricular trabeculae. Values indicate mean ± S.E.M. (n = 5). Statistical significance of differences between values was assessed using the paired Student’s t-test: †P < 0.05; ‡P < 0.01; *P < 0.001

| | Relaxed | | | Active | | | Rigor | | |
|---|---|---|---|---|---|---|---|---|---|
| | $\langle P_{2d} \rangle$ | $\langle P_{2} \rangle$ | $\langle P_{4} \rangle$ | $\langle P_{2d} \rangle$ | $\langle P_{2} \rangle$ | $\langle P_{4} \rangle$ | $\langle P_{2d} \rangle$ | $\langle P_{2} \rangle$ | $\langle P_{4} \rangle$ |
| BSR-cRLC-A | 0.7033 ± 0.0101 | 0.0011 ± 0.0074 | 0.1520 ± 0.0124 | 0.6922 ± 0.0185 | 0.0241 ± 0.0109† | 0.1399 ± 0.0090 | 0.6774 ± 0.0177 | 0.1774 ± 0.0100* | 0.1449 ± 0.0233 |
| BSR-cRLC-BC | 0.8165 ± 0.0112 | -0.0046 ± 0.0057 | -0.0404 ± 0.0066 | 0.8095 ± 0.0143 | 0.0592 ± 0.0074* | -0.0297 ± 0.0100† | 0.8180 ± 0.0137 | 0.0386 ± 0.0139* | -0.0079 ± 0.0109* |
| BSR-cRLC-C | 0.5985 ± 0.0094 | -0.0636 ± 0.0084 | 0.1944 ± 0.0166 | 0.5773 ± 0.0079 | -0.0637 ± 0.0099 | 0.2283 ± 0.0107* | 0.5851 ± 0.0158 | -0.1721 ± 0.0246 | 0.2501 ± 0.0313* |
| BSR-cRLC-D | 0.6772 ± 0.0213 | 0.0087 ± 0.0077 | 0.0810 ± 0.0335 | 0.6615 ± 0.0257 | -0.0198 ± 0.0043* | 0.1382 ± 0.0376* | 0.6769 ± 0.0191 | -0.1008 ± 0.0105* | 0.0889 ± 0.0226 |
| BSR-cRLC-E | 0.6424 ± 0.0153 | 0.1359 ± 0.0127 | 0.1259 ± 0.0175 | 0.6415 ± 0.0163 | 0.1003 ± 0.0138‡ | 0.1225 ± 0.0156 | 0.6637 ± 0.0166 | -0.0508 ± 0.0163* | 0.0407 ± 0.0169* |
| BSR-cRLC-F | 0.8972 ± 0.0235 | 0.0275 ± 0.0028 | -0.0426 ± 0.0196 | 0.8922 ± 0.0240 | 0.0117 ± 0.0068† | -0.0546 ± 0.0167 | 0.8948 ± 0.0217 | -0.1025 ± 0.0153* | -0.0416 ± 0.0199 |
| BSR-cRLC-G | 0.6380 ± 0.0096 | 0.0155 ± 0.0057 | -0.0171 ± 0.0222 | 0.6215 ± 0.0124 | 0.0220 ± 0.0074 | 0.0038 ± 0.0257† | 0.6227 ± 0.0104 | 0.0072 ± 0.0093 | 0.0019 ± 0.0281 |
| BSR-cRLC-FG | 0.6541 ± 0.0084 | 0.0347 ± 0.0072 | 0.0982 ± 0.0110 | 0.6412 ± 0.0097 | 0.0262 ± 0.0051† | 0.1172 ± 0.0085 | 0.6371 ± 0.0117 | -0.0026 ± 0.0075* | 0.0919 ± 0.0119 |

**Table S3.** Mean orientation $\theta_{ME}$ [°] of RLC probes with respect to the filament axis and standard deviation $\sigma_{ME}$ of one - dimensional maximum entropy distributions calculated from the measured order parameters $<P_2>$ and $<P_4>$

| | Relaxed | | Active | | Rigor | |
|---|---|---|---|---|---|---|
| | $\theta_{ME}$ | $\sigma_{ME}$ | $\theta_{ME}$ | $\sigma_{ME}$ | $\theta_{ME}$ | $\sigma_{ME}$ |
| BSR-cRLC-A | 57.3 ± 0.4 | 26.4 ± 0.3 | 56.2 ± 0.6 | 26.3 ± 0.3 | 48.6 ± 0.5* | 26.7 ± 0.7 |
| BSR-cRLC-BC | 56.8 ± 0.8‡ | 20.1 ± 0.5 | 54.6 ± 0.2‡ | 20.7 ± 0.7 | 55.4 ± 0.1† | 21.3 ± 0.7 |
| BSR-cRLC-C | 60.7 ± 0.4 | 26.8 ± 0.5 | 60.8 ± 0.5 | 27.7 ± 0.4 | 66.6 ± 1.4‡ | 25.8 ± 0.9 |
| BSR-cRLC-D | 56.9 ± 0.4 | 24.1 ± 0.8 | 58.4 ± 0.1‡ | 25.4 ± 1.0 | 62.1 ± 0.3* | 21.2 ± 1.6* |
| BSR-cRLC-E | 50.3 ± 0.8‡ | 26.3 ± 0.5 | 52.4 ± 0.7‡ | 26.6 ± 0.7 | 59.8 ± 0.9‡ | 22.2 ± 0.7* |
| BSR-cRLC-F | 55.5 ± 0.2 | 20.7 ± 0.7 | 56.3 ± 0.3† | 20.1 ± 0.6 | 61.8 ± 0.8† | 18.3 ± 0.5* |
| BSR-cRLC-G | 56.2 ± 0.3 | 21.4 ± 0.7 | 55.9 ± 0.4 | 22.2 ± 0.8 | 56.7 ± 0.5 | 21.9 ± 0.8 |
| BSR-cRLC-FG | 55.5 ± 0.4 | 24.0 ± 0.3 | 56.0 ± 0.3 | 25.7 ± 0.3 | 57.4 ± 0.4† | 24.6 ± 0.3 |

Values indicate mean ± SEM (n=5). Statistical significance of differences between values was assessed using the paired student's t-test: †$P < 0.05$; ‡$P < 0.01$; *$P < 0.001$

## Supporting References

1. Risal, D., S. Gourinath, D. M. Himmel, A. G. Szent-Gyorgyi, and C. Cohen. 2004. Myosin subfragment 1 structures reveal a partially bound nucleotide and a complex salt bridge that helps couple nucleotide and actin binding. *P Natl Acad Sci USA* 101:8930-8935.
2. Brown, J. H., V. S. Kumar, E. O'Neall-Hennessey, L. Reshetnikova, H. Robinson, M. Nguyen-McCarty, A. G. Szent-Gyorgyi, and C. Cohen. 2011. Visualizing key hinges and a potential major source of compliance in the lever arm of myosin. *P Natl Acad Sci USA* 108:114-119.
3. Rayment, I., W. R. Rypniewski, K. Schmidt-Base, R. Smith, D. R. Tomchick, M. M. Benning, D. A. Winkelmann, G. Wesenberg, and H. M. Holden. 1993. Three-dimensional structure of myosin subfragment-1: a molecular motor. *Science* 261:50-58.
4. Lawson, J. D., E. Pate, I. Rayment, and R. G. Yount. 2004. Molecular dynamics analysis of structural factors influencing back door pi release in myosin. *Biophys J* 86:3794-3803.
5. Kinoshita, E., E. Kinoshita-Kikuta, K. Takiyama, and T. Koike. 2006. Phosphate-binding tag, a new tool to visualize phosphorylated proteins. *Mol. Cell Proteomics* 5:749-757.
6. Gourinath, S., D. M. Himmel, J. H. Brown, L. Reshetnikova, A. G. Szent-Gyorgyi, and C. Cohen. 2003. Crystal structure of scallop Myosin s1 in the pre-power stroke state to 2.6 a resolution: flexibility and function in the head. *Structure* 11:1621-1627.
7. Debreczeni, J. E., L. Farkas, V. Harmat, C. Hetenyi, I. Hajdu, P. Zavodszky, K. Kohama, and L. Nyitray. 2005. Structural evidence for non-canonical binding of Ca2+ to a canonical EF-hand of a conventional myosin. *J Biol Chem* 280:41458-41464.
8. Romano, D., B. D. Brandmeier, Y. B. Sun, D. R. Trentham, and M. Irving. 2012. Orientation of the N-terminal lobe of the myosin regulatory light chain in skeletal muscle fibers. *Biophys J* 102:1418-1426.
9. Brack, A. S., B. D. Brandmeier, R. E. Ferguson, S. Criddle, R. E. Dale, and M. Irving. 2004. Bifunctional rhodamine probes of Myosin regulatory light chain orientation in relaxed skeletal muscle fibers. *Biophys J* 86:2329-2341.
