# Supplementary material for: Orientation of the N- and C-Terminal Lobes of the Myosin Regulatory Light Chain in Cardiac Muscle
Source: Biophys J. 2015 Jan 20;108(2):304–14. doi: 10.1016/j.bpj.2014.11.049 (PMC4302210; doi:10.1016/j.bpj.2014.11.049)
Supplement: Document S2. Article plus Supporting Material [file mmc2.pdf]

Article

Orientation of the N- and C-Terminal Lobes of the Myosin Regulatory Light Chain in Cardiac Muscle

Thomas Kampourakis,[1] Yin-Biao Sun,[1] and Malcolm Irving[1,*]
[1]Randall Division of Cell and Molecular Biophysics and British Heart Foundation Centre of Research Excellence, King's College London, London, United Kingdom

ABSTRACT The orientations of the N- and C-terminal lobes of the cardiac isoform of the myosin regulatory light chain (cRLC) in the fully dephosphorylated state in ventricular trabeculae from rat heart were determined using polarized fluorescence from bifunctional sulforhodamine probes. cRLC mutants with one of eight pairs of surface-accessible cysteines were expressed, labeled with bifunctional sulforhodamine, and exchanged into demembranated trabeculae to replace some of the native cRLC. Polarized fluorescence data from the probes in each lobe were combined with RLC crystal structures to calculate the lobe orientation distribution with respect to the filament axis. The orientation distribution of the N-lobe had three distinct peaks (N1–N3) at similar angles in relaxation, isometric contraction, and rigor. The orientation distribution of the C-lobe had four peaks (C1–C4) in relaxation and isometric contraction, but only two of these (C2 and C4) remained in rigor. The N3 and C4 orientations are close to those of the corresponding RLC lobes in myosin head fragments bound to isolated actin filaments in the absence of ATP (in rigor), but also close to those of the pair of heads folded back against the filament surface in isolated thick filaments in the so-called J-motif conformation. The N1 and C1 orientations are close to those expected for actin-bound myosin heads with their light chain domains in a pre-powerstroke conformation. The N2 and C3 orientations have not been observed previously. The results show that the average change in orientation of the RLC region of the myosin heads on activation of cardiac muscle is small; the RLC regions of most heads remain in the same conformation as in relaxation. This suggests that the orientation of the dephosphorylated RLC region of myosin heads in cardiac muscle is primarily determined by an interaction with the thick filament surface.

INTRODUCTION

The globular head of the myosin molecule, also called subfragment-1 or S1, is the molecular motor that drives muscle contraction, performing mechanical work in cyclical interactions with actin in the thin filament coupled to hydrolysis of ATP (1–3). In each interaction, the myosin motor is thought to undergo a conformational change, the working stroke, which is associated with axial tilting of its light chain domain (LCD; Fig. 1)—containing the myosin regulatory light chain (RLC) and essential light chain (ELC)—with respect to the actin-attached catalytic domain (CD) (4–7). Tilting of the LCD is generally assumed to be linked to the thick filament backbone via the coiled-coil subfragment-2 (S2) domain of myosin, thereby generating relative sliding of the thick filaments with respect to the interdigitating thin filaments.

According to the above model, the LCD should be relatively rigid, so that it efficiently amplifies small conformational changes in the CD, and the S1/S2 junction should be a free pivot, allowing a change in orientation of the whole LCD during the working stroke as the filaments slide. However, neither of these postulates has strong experimental support. X-ray crystallography (8–10) of isolated myosin fragments has revealed multiple LCD conformations, suggesting there are internal hinges between the RLC and ELC, between the N- and C-lobes of the RLC, and near the CD/LCD junction. Although it is clear at low resolution that there is significant flexibility between the S1 and S2 regions of myosin (11), the conventional definition of the junction between these two domains is based on proteolytic susceptibility rather than on high-resolution structural data. Thus, the possibility that the functional lever arm might be composed of only part of the LCD, and that the functional pivot between the myosin motor and the thick filament might be within the LCD rather than at the S1-S2 junction, has not been excluded. This has fundamental implications for both molecular models of contractility and the regulatory role of the RLC in striated muscle.

The interaction between myosin and actin in striated muscle is primarily regulated by transient binding of Ca^{2+} ions to the troponin/tropomyosin complex in the thin filament (12,13), although phosphorylation of thin and thick filament proteins including the RLC are implicated in physiological and pathological modulation of contractility on slower timescales (14–17). In the heart, decreased RLC phosphorylation is associated with impaired contractile function and cardiac

Submitted June 16, 2014, and accepted for publication November 10, 2014.
*Correspondence: malcolm.irving@kcl.ac.uk
This is an open access article under the CC BY license (http://creativecommons.org/licenses/by/3.0/).
Editor: Bernhard Brenner.
© 2015 The Authors
0006-3495/15/01/0304/11 $2.00
http://dx.doi.org/10.1016/j.bpj.2014.11.049

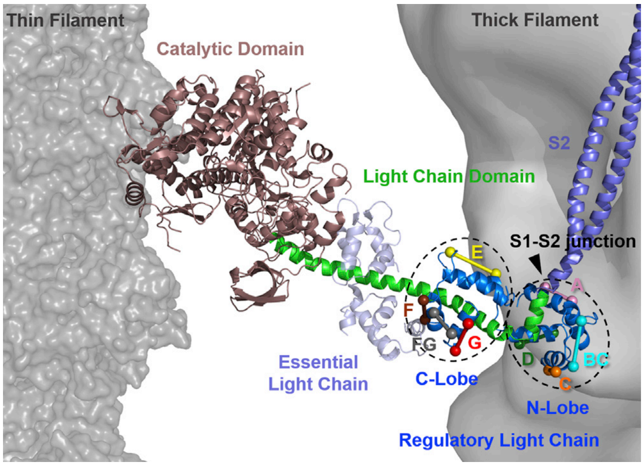

FIGURE 1 Conformation of myosin domains in muscle and regulatory light chain probes. The regulatory light chain (*blue*) and essential light chain (*light purple*) are shown bound to the light chain domain of the myosin heavy chain (*green*) in the rigor actin-myosin head complex, with the catalytic domain in brown (5,41). BSR-probes were introduced on RLC helix-A (*pink*), cross-linking helices *B* and *C* (*cyan*), *C* (*orange*), *D* (*dark green*), *E* (*yellow*), *F* (*brown*), *G* (*red*), and cross-linking helices *F* and *G* (*gray*). The Cβ-atoms (or Cα-atoms in case of glycine residues) of mutated residues are shown as colored spheres and the expected probe dipole orientations are indicated by sticks. Surface views of the thin and thick filaments (*gray*) are from (61) and (43), respectively. The myosin subfragment-2 structure (*violet*) is from human cardiac myosin S2Δ (PDB 2FXO (52)).

hypertrophy in animal models (18), and contractile regulation by another thick filament component—myosin binding protein-C (MyBP-C)—may also be partly mediated by its interaction with the RLC (19). The high frequency of familial hypertrophic cardiomyopathy mutations associated with cardiac RLC (20) further emphasizes its functional importance.

To better understand the physiological function of the RLC in the context of both the tilting lever arm model and the regulation of contractility, we measured the orientation of its N- and C-lobes in the native environment of heart muscle cells. Although it is technically challenging to make molecular structural measurements at the whole cell level, this approach has the important advantage of preserving both the intra- and intermolecular interactions between myosin domains in a native thick filament and the native interaction between myosin and actin in the filament array at physiological concentrations of ATP and calcium. We measured RLC lobe orientation in ventricular trabeculae using sets of site-specific bifunctional sulforhodamine (BSR) probes cross-linking genetically introduced pairs of cysteine residues on the surface of the RLC (21–24) (Fig. 1). The orientation of each BSR probe was determined in situ from the polarization of its fluorescence, and the data from the set of probes on each RLC lobe were combined to calculate the orientation of the lobe with respect to the filament axis. Thus, we measured RLC N- and C-lobe orientation in the relaxed (low calcium or diastolic) state in which myosin is detached from actin, in the nucleotide-free rigor state in which the heads are expected to be strongly bound to actin, and during active isometric contraction at physiological ATP concentration and micromolar calcium concentration, corresponding to the systolic phase of the cardiac cycle.

MATERIALS AND METHODS

Preparation and characterization of BSR-cRLCs

Mutants of the human ventricular cRLC (UniProtKB entry: MLRV_HUMAN; P10916) with pairs of cysteines introduced at positions 27 and 34 on helix-A, 64 and 71 on helix-C, 80 and 88 on helix-D, 54 and 63 cross-linking helices B and C, 97 and 110 on helix-E, 131 and 138 on helix-G, 117 and 125 on helix-F or 120 and 136 cross-linking helices F and G (Fig. 1) were obtained by site-directed mutagenesis. The mutants were expressed in BL21(DE3) cells (Stratagene) as N-terminal fusion proteins with a Histidine tag and TEV protease site from a pET6a vector.

After removal of the N-terminal tag sequence by TEV protease, each of the cRLC double-cysteine mutants was labeled with BSR-I_2 (Invitrogen, B-10621) and purified as described (23). The labeled BSR-cRLC-conjugates are referred to as BSR-cRLC-X, where X indicates the cRLC helix to which the BSR probe is attached or the pair of helices cross-linked by the probe. Bifunctional labeling produces diastereoisomers; these could sometimes be separated by high-performance liquid chromatography, but mixed isomers were used for the experiments reported below. The two diastereoisomers are expected to have the same orientation with respect to the backbone of the labeled protein (21,23–25). Purity of the conjugates was characterized by high-performance liquid chromatography (Agilent 1200 Series; Agilent Technologies, Stockport, Cheshire, UK) and electrospray mass spectrometry (Hewlett Packard Series 1100 LC/MS). BSR-cRLCs were obtained on the 1–10 mg scale at 94–96% purity. Measured (calculated) masses (Daltons) of the BSR-cRLCs A, BC, C, D, E, F, G, and FG were 19583.2 (19583.2), 19629.9 (19626.2), 19554.0 (19556.0), 19581.2 (19582.6), 19625.3 (19627.0), 19595.2 (19596.8), 19611.2 (19613.0), and 19540.5 (19541.6), respectively. Circular dichroism (CD) spectra were measured in 10 mM phosphate buffer at pH 7.4 on an Applied Photophysics Chirascan Plus spectrometer.

Determination of endogenous cRLC phosphorylation level

For determination of the extent of cRLC phosphorylation, skinned trabeculae preparations (see below) were dissolved in SDS-PAGE sample buffer and run on 15% SDS-acrylamide (30:1 acrylamide:bis-acrylamide) gels (26) containing 50 μM Phos-tag-Acrylamide (NARD Institute, Hyogo, Japan) (27) and 100 μM $MnCl_2$. After electrophoresis, gels were equilibrated for 15 min in transfer buffer (25 mM Tris, pH 8.3, 192 mM glycine, 20% (v/v) methanol) containing 1 mM EDTA and then washed three times in transfer buffer without EDTA. Gels were blotted for 1 h at 50 mA in transfer buffer onto nitrocellulose membranes (Bio-Rad Laboratories, Hemel Hempstead, UK) using a Trans-Blot SD Semi-Dry Electrophoretic Transfer Cell (Bio-Rad). Postblotting, membranes were blocked for 1 h at room temperature in Tris-buffered saline containing 0.05% (v/v) Tween-20 (TBS-T, Calbiochem, Nottingham, UK) containing 5% (w/v) nonfat dried milk powder. The blocked membranes were briefly washed with TBS-T and then incubated overnight at 4°C with primary antibody against cRLC (rabbit monoclonal antimyosin light chain 2, ABCAM, ab92721, Cambridge, UK) in a 1:10000 dilution in TBS-T containing 2.5% (w/v) nonfat dried milk powder. Membranes were washed with TBS-T and incubated for 1 h at room temperature with secondary antibody (1:1000 dilution, HRP-conjugated donkey anti-rabbit IgG, NA934V, GE

Healthcare, Buckinghamshire, UK) in TBS-T containing 1% (w/v) nonfat dried milk powder. Blots were washed in TBS-T, immersed in ECL Plus reagent (GE Healthcare), and bands visualized by developing the blot with BioMax MR film (Kodak, Leicestershire, UK).

Reconstitution of BSR-cRLCs into ventricular trabeculae

Wistar rats (male, 200–250 g) were sacrificed by cervical dislocation (Schedule 1 procedure in accordance with UK Animals Scientific Procedures Act, 1986). The hearts were removed immediately and rinsed free of blood in Krebs-Henseleit solution (Sigma, K3753, Gillingham, UK) containing: 118 mM NaCl, 24.8 mM $NaHCO_3$, 1.8 mM Na_2HPO_4, 1.18 mM $MgSO_4$, 4.75 mM KCl, 2.54 mM $CaCl_2$, and 10 mM glucose bubbled with 95% O_2-5% CO_2, pH 7.4 at 20°C. Suitable trabeculae (free running, unbranched, diameter $\leq$250 μm) were dissected from the right ventricle in Krebs solution containing 25 mM 2,3-butanedione-monoxime, demembranated in relaxing solution (25 mM imidazole, 5 mM MgATP, 1 mM free Mg^{2+}, 10 mM EGTA, 1 mM dithiothreitol (DTT), and 0.1% (v/v) protease inhibitor cocktail (P8340, Sigma); ionic strength adjusted to 200 mM with KPropionate; pH 7.1 at 20°C) containing 1% (v/v) Triton X-100 for 60 min on ice and stored in relaxing solution containing 50% (v/v) glycerol at −20°C for experiments. Trabeculae were used for experiments within 2 days of dissection.

Demembranated trabeculae were mounted via aluminum T-clips at sarcomere length 2.1 μm between a force transducer (Kronex Technologies, AE 801, Oakland, CA) and a fixed hook in a 60 μl glass trough containing relaxing solution. The cross-sectional areas of trabeculae were calculated from the width assuming circular profile. Trabeculae were activated in 25 mM imidazole, 5 mM MgATP, 1 mM free Mg^{2+}, 10 mM CaEGTA, 1 mM DTT (ionic strength adjusted to 200 mM with KPropionate; pH 7.1 at 20°C; activating solution) preceded by 2 min incubation in preactivating solution (as relaxing solution except EGTA reduced to 0.2 mM), and the maximal isometric force before cRLC exchange was recorded. BSR-cRLCs were exchanged into trabeculae by a protocol modified from that used previously for skeletal muscle (28). In most experiments BSR-cRLCs were introduced by incubating trabeculae for 30 min at 22°C in an EDTA-rigor-extract solution (10 mM K_2PO_3H, pH 7.1, 20 mM EDTA, 115 mM KPropionate) containing 0.5 mg/ml BSR-cRLC and 10 mM DTT. The trabeculae were then washed for 5 min in relaxing solution and subsequently bathed in relaxing solution containing 0.5 mg/ml recombinant human cardiac troponin C and troponin complex (kindly provided by Dr. Mitla Garcia) for 15 min and 1 h, respectively. In some control experiments the native cRLC was extracted in *trans*-1,2-cyclohexanediamine-*N,N,N′,N′*-tetraacetic acid (CDTA)-rigor solution (composition in mM: 5 CDTA, 50 KCl, 40 Tris-HCl pH 8.4, 0.1% (v/v) Triton X-100) (20) followed by reconstitution with 40 μM BSR-cRLC in relaxing solution (composition in mM: 25 Imidazole, 15 Na_2Creatine phosphate (Na_2CrP), 78.4 KPropionate (KPr), 5.65 Na_2ATP, 6.8 $MgCl_2$, 10 K_2EGTA, 1 DTT, pH 7.1) replacing over 50% of the endogenous cRLC. Trabeculae were then washed in relaxing solution for 30–45 min, the sarcomere length adjusted to 2.1 μm and activated again. The maximal isometric force was recorded, and trabeculae showing <75% force recovery compared to that before cRLC exchange were discarded.

The extent of cRLC exchange was determined by comparing the fluorescence intensity of the relaxed trabeculae with that of a known concentration of the same BSR-cRLC in a 50 μm path-length cuvette mounted in the same place on the experimental setup. The relatively mild cRLC exchange conditions used here resulted in replacement of ~12% ± 3% (mean ± SD, $n =$ 5) of the endogenous cRLCs by BSR-cRLCs. The average isometric force after cRLC exchange was 88% ± 13% (mean ± SD, $n = 30$) of that before exchange, and the extent of force recovery was the same within experimental variability for all eight BSR-cRLCs, and consistent with published data for RLC exchange in skeletal muscle (22–24,28,29), indicating that the small decrease in force is a nonspecific effect of the RLC exchange protocol. The CDTA extraction/cRLC reconstitution protocol resulted in replacement of 50% ± 5% (mean ± SE, n = 7) replacement of the cRLCs by BSR-cRLC, with a similar force recovery, 87 ± 6% (mean ± SE, $n = 7$). Incorporation of BSR-cRLCs into the A-bands of the trabecular sarcomeres was confirmed by confocal microscopy using A.1025 antibody against myosin heavy chain as a counter-stain (Fig. S2 *A* in the Supporting Material).

Fluorescence polarization measurements

Activation protocols were as described for polarized fluorescence experiments with probes on cTnC in trabeculae (30). Polarized fluorescence intensities were measured as described previously for skeletal muscle fibers (23). Fluorescence emission from BSR-cRLCs in trabeculae was collected by a 0.25 N.A. objective using excitation light beams either in line with or at 90° to the emission path. The polarization of the excitation and emitted beams was set either parallel or perpendicular to the trabecular axis, allowing determination of the three-order parameters, $\langle P_{2d} \rangle$, $\langle P_2 \rangle$ and $\langle P_4 \rangle$ that describe the orientations of the probe dipoles with respect to the trabecular axis (31). One-dimensional maximum entropy (ME) distributions were calculated from the $\langle P_2 \rangle$ and $\langle P_4 \rangle$ values for each probe and condition and expressed as the mean θ_{ME} and standard deviation σ_{ME} of the resulting ME distribution for each probe (32). The orientations of the cRLC N- and C-lobes were calculated by two-dimensional ME analysis, combining the data from four BSR-cRLCs in each case (33). The orientation of each probe dipole in the coordinate frame of a given crystallographic structure of the N-lobe was calculated using a coordinate frame defined by the D- and B-helices (Fig. S3). The orientation of the N-lobe in the laboratory frame was then described by the angles β_{DB} and γ_{DB}, where β_{DB} is the angle between the D-helix and the trabecular/thin filament axis, and γ_{DB} describes the rotation of the lobe around the D-helix, with $\gamma_{DB} = 0°$ when the plane containing the D- and B-helices coincides with that containing the D-helix and filament axis. A similar approach was used for probes in the C-lobe, using the E- and G-helices as the corresponding reference axes for β_{EG} and γ_{EG}, respectively (Fig. S3). An increase in either γ indicates a counterclockwise rotation of each lobe viewed from the + end of the D- or E-helix.

For the N-lobe of scallop myosin S1 in the nucleotide-free state (Protein Data Bank (PDB) entry 1SR6 (34)), the D-helix orientation was defined by the vector joining the midpoints of the Cα atoms of residue pairs Thr-70/Phe-72 and Ser-74/Phe-76 oriented toward increasing residue number, and that of the B-helix by a vector joining the midpoints of Cα atoms of the residue pairs Asp-39/Lys-41 and Ile-43/Glu-45. In the C-lobe, the E-helix orientation was defined by the vector joining the Cα atoms of residue pairs Glu-87/Ile-89 and Asn-91/Phe-93, and the G-helix by a vector joining the midpoints of Cα atoms of the residue pairs Asp-123/Met-125 and Met-127/Phe-129. The orientation of each probe dipole, calculated in the local coordinate frame under the assumption that the probe dipole is parallel to the BSR attachment points, is described as (θ,ϕ), where θ is the angle between the probe dipole and the D-helix in the N-lobe, and between the probe dipole and the E-helix in the C-lobe. ϕ is defined as the angle between the D-helix/dipole plane and the D-helix/B-helix plane for the N- lobe, and between the E-helix/dipole plane and the E-helix/G-helix plane for the C-lobe. An increase in ϕ indicates a counterclockwise rotation of the probe viewed from the + end of the D- or E-helix.

Probe dipole orientations were calculated from six different crystal structures of the RLC from five isoforms (PDB entries 2MYS, 1SR6, 3PN7 (molecules 1 and 2), 1QVI, and 2BL0) in the coordinate frames defined above, by aligning the sequences to define the helix orientations and probe attachment points for residue numbers corresponding to those given above for human ventricular cRLC (Fig. S3). The average probe dipole orientations (θ,ϕ) and standard deviations for BSR-cRLCs A, BC, C, D, E, F, G, and FG calculated for the six structures are (55.8° ± 4.5°, −85.1° ± 5.6°), (102.2° ± 5.1°, −154.5° ± 6.8°), (80.0° ± 10.4°, 124.8° ± 5.0°), (166.2° ± 5.7°, −93.9° ± 32.9°), (157.3° ± 5.2°, −22.1° ± 21.8°), (78.2° ± 5.6°,

160.0° ± 4.3°), (82.2° ± 2.7°, -5.6° ± 3.4°), and (48.3° ±6.7°, -110.1° ± 10.9°), respectively (Fig. S3).

RESULTS

Preparation and incorporation of BSR-cRLCs into skinned cardiac trabeculae

Eight double-cysteine mutants of the human ventricular isoform of the RLC (cRLC) were designed for bifunctional attachment of a rhodamine probe in a range of orientations on the surface of either the N- or C-lobe of cRLC (Fig. 1). Expression, BSR-labeling, purification, and characterization of these cRLCs are described in the Materials and Methods. The resulting BSR-cRLC conjugates are referred to as BSR-cRLC-X, where X indicates the cRLC helix to which the BSR probe is attached or the pair of helices cross-linked by the probe (Fig. 1). The effects of mutagenesis and BSR labeling on the structure of the cRLC were assessed by far-ultraviolet CD spectroscopy. CD spectra for wild-type cRLC and BSR-cRLC-G (Fig. S1) show typical bands for α-helical proteins (35) with α-helical contents of 16% and 18% for the wild-type cRLC and BSR-cRLC-G, respectively, in agreement with published results for isolated RLC in solution (20,36).

In most experiments BSR-cRLCs were introduced into skinned trabeculae from rat right ventricle using milder cRLC exchange conditions (see Materials and Methods) than those used previously in skeletal muscle (22,23,28), because recent results showed that the milder RLC exchange protocol gives better preservation of thick filament structure (37). In ventricular trabeculae this protocol resulted in replacement of ~12% of the native cRLC by BSR-cRLCs, which were mainly localized in the myosin-containing A-band of the sarcomere as expected (Fig. S2 *A*). Moreover BSR-cRLCs seemed to be confined to the inner two-thirds of the A-band, suggesting preferential exchange of the endogenous cRLC in the C-zone of the sarcomere, which contains MyBP-C. A small fraction (<5%) of the BSR-cRLC localized to the sarcomeric Z-disk and M-band. Previous studies using RLCs labeled with bifunctional rhodamine at many different sites in skeletal muscle (22–24,29) showed that introduction of the probe did not affect RLC function. To check whether this is also the case for cRLC in cardiac trabeculae, we used a CDTA exchange protocol that resulted in the replacement of ~50% of native cRLC by BSR-labeled cRLC (Table S1). Isometric force recovery after such exchange was the same as in the milder protocol that produced 12% cRLC exchange, indicating that mutagenesis and BSR labeling does not affect cRLC function.

The endogenous cRLCs in the skinned cardiac trabeculae were fully dephosphorylated in the conditions of our experiments (Fig. S2 *B*), in contrast with the 0.4–0.5 mol P_i/mol cRLC phosphorylation level reported for myocardium in vivo (15). This difference may be associated with the protocols used for preparing the trabeculae, or the region of the heart from which they were obtained (38). cRLCs were also fully dephosphorylated in control experiments on unskinned trabeculae, and on trabeculae in which butanedione monoxime, a drug that has been suggested to act as a chemical phosphatase (39,40), was omitted from all solutions.

Orientation of the BSR-cRLC probes in cardiac muscle cells

The orientation of the BSR-cRLC probes with respect to the filament or trabecular axis was calculated from the polarization of the fluorescence from each probe after exchange into trabeculae. These measurements give information about $\cos^2\theta$ and $\cos^4\theta$, where θ is the angle between the probe dipole, which is approximately parallel to the line joining the two cysteines where the probe is attached, and the filament axis (21,31). This orientation information is conventionally presented in terms of the order parameters $\langle P_{2d} \rangle$, $\langle P_2 \rangle$, and $\langle P_4 \rangle$ that can be obtained directly from the polarized fluorescence intensities, where $\langle P_{2d} \rangle$ gives information about independent mobility of the probes with respect to the RLC on the subnanosecond timescale, and $\langle P_2 \rangle$ and $\langle P_4 \rangle$ represent the time-averaged orientation of the probe on slower timescales (25,31). These data are presented in Table S2. As reported previously for bifunctional rhodamine probes on the RLC in skeletal muscle fibers (22–24), $\langle P_{2d} \rangle$ has a characteristic value for each labeling site but for any given site is approximately independent of the contractile state of the muscle cells (Table S2). All the order parameters ($\langle P_{2d} \rangle$, $\langle P_2 \rangle$ and $\langle P_4 \rangle$) obtained with the mild RLC exchange protocol used in the experiments reported below were similar to those obtained with the CDTA extraction protocol that resulted in 50% cRLC exchange (Table S1), indicating that the RLC exchange protocol does not alter probe orientation or its dynamics.

To provide a more physically accessible description of the time-averaged orientation of the probe dipoles with respect to the actin filament or trabecular axis, we calculated a one-dimensional ME distribution (32) for each probe and condition, and the mean θ_{ME} and standard deviation σ_{ME} of these distributions are shown in Fig. 2. The relatively high degree of disorder of RLC orientations in all conditions studied means that the θ_{ME} values cluster around 50–70° and the σ_{ME} values are mostly >20°, for both the N-lobe probes (Fig. 2 *A*) and C-lobe probes (Fig. 2 *B*). This analysis does not give useful information about the shape of the orientation distributions, which are necessarily simplified because only two orientation parameters are measured for each probe. Thus, it does not capture the full complexity of the orientation distributions, for example the existence of multiple populations of probes with distinct orientations, but does show trends in the orientation changes for each probe with high angular resolution, because the θ_{ME} and σ_{ME} values are determined precisely. These trends are

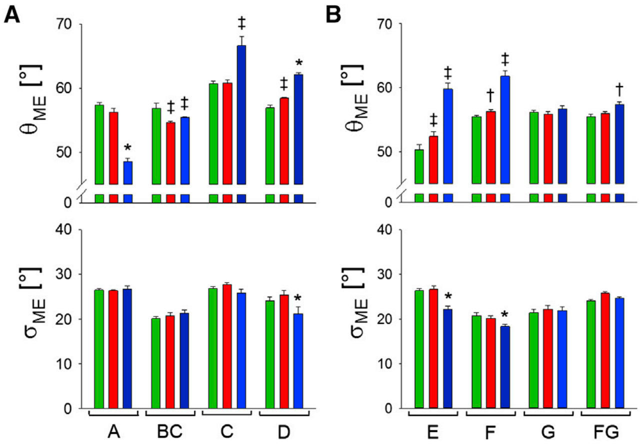

FIGURE 2 Orientation parameters of BSR probes on the N-lobe (*A*) and C-lobe (*B*) of the RLC in ventricular trabeculae. θ_{ME} is the mean and σ_{ME} the standard deviation of a one-dimensional maximum entropy orientation distribution for each probe in relaxation (*green*), active contraction (*red*), and rigor (*blue*). Statistical significance of differences between values was assessed using the paired Student's *t*-test: †P < 0.05; ‡P < 0.01; *P < 0.001.

similar to those reported previously for RLCs labeled with bifunctional rhodamine in skeletal muscle fibers (21,24), namely the tendency for the mean probe orientation θ_{ME} for active contraction (*red bars*) to be intermediate between that for relaxation (*green*) and rigor (*blue*), but closer to the relaxed value, and for the standard deviation σ_{ME} to be either similar in the three states or lower in rigor. The mean orientation θ_{ME} was significantly different ($P < 0.05$) between rigor and relaxation for seven of the eight probes, and between relaxation and active contraction for four probes, two in the N-lobe and two in the C-lobe.

Orientation of the cRLC N-lobe

More detailed information about the orientation distribution of the N-lobe of the RLC with respect to the filament axis was obtained by combining the $\langle P_2 \rangle$ and $\langle P_4 \rangle$ data from multiple probes using two-dimensional ME analysis (31,33). The resulting ME distributions are the smoothest distributions of lobe orientations consistent with the eight order parameters measured for the N-lobe probes, and give an exact fit to those parameters. The orientation distributions were described using an internal reference frame defined by the D- and B-helices of the RLC N-lobe, written as $(\beta,\gamma)_{DB}$, where β_{DB} is the angle between the D-helix and the filament axis and γ_{DB} describes the rotation of the lobe around the D-helix using the B-helix to define $\gamma_{DB} = 0$ (see Materials and Methods). The calculated orientation distribution depends on the relative orientation of the four probes, and therefore on the chosen reference structure of the RLC lobe. To characterize this dependence we repeated the calculations using six crystallographic structures of the RLC lobes of myosins from skeletal and smooth muscles from a wide range of species. (Currently there is no structure of the RLC region of a cardiac myosin in the PDB.) The backbone fold of the RLC is well conserved between published structures, and the orientations of the cysteine pairs used for probe attachment varied by typically $\pm 10°$ (Fig. S3). The results presented here were calculated using the reference frame from PDB entry 1SR6 (34); those obtained using other structures are described and compared in the Supporting Material (Fig. S4)

ME distributions for the RLC N-lobe are shown as contour plots in Fig. 3, *A–C*, with hotter colors denoting a higher probability that the N-lobe is at that orientation. The plots are limited to the range $-90° < \gamma < 90°$ because dipole probes cannot distinguish between orientations (β, γ) and $(180° - \beta, 180° + \gamma)$. This leads to wrapping of features on the upper left boundary of the plot to the lower right and vice versa.

The $(\beta,\gamma)_{DB}$ N-lobe orientation maps show three peaks for all conditions studied, with approximate center angles $(\beta,\gamma)_{DB} = (60°, -30°)$, $(105°, 70°)$, and $(135°, -45°)$, which we refer to as N1, N2, and N3, respectively. The distributions for relaxation and active contraction (Fig. 3, *A* and *B*) are very similar, as expected from the similarity of the corresponding θ_{ME} and σ_{ME} values for individual probes (Fig. 2 *A*). The N1 peak has a slightly higher peak β in active contraction. In rigor (Fig. 3 *C*), the N1 and N3 peaks partially merge in β.

The RLC N-lobe orientations corresponding to peaks N1, N2, and N3 are shown graphically in Fig. 3 *D* in relation to the coiled-coil subfragment-2 (*light blue*) to which pairs of myosin heads, and thus pairs of cRLC N-lobes, are attached in situ. For the N1 peak the heavy chain hook helix (*green*), which forms the backbone of the N-lobe, is almost parallel to the filament axis (the axial angle between the helix and the filament axis is 8°), whereas for N2 and N3 it is almost perpendicular (axial angles 108 and 83°, respectively). N1/N2 and N2/N3 cRLC dimers would allow a separation between the two cRLC N-lobes of a single myosin molecule, but an N1/N1 combination (not shown), with both hook helices parallel to the S2 coiled-coil, could only be accommodated by separation of the two chains of the S2 coiled-coil at its N-terminus.

The RLC N-lobe orientation corresponding to the N1 peak lies between that in the canonical structure of isolated heads from chicken skeletal muscle myosin (2MYS) bound to actin filaments in the absence of ATP, i.e., in rigor (41), (Fig. 3 *C*, *green triangle*) and that expected for the prepowerstroke or ADP.Pi state (Fig. 3 *A*, *pink triangle*), modeled using the smooth muscle myosin head structure (1BR1) with its catalytic domain fitted to that of the actin-bound 2MYS, and the RLC region from 2MYS added by superimposing the ELC regions as in (21). The N3 orientation is close to those produced when the catalytic domains of scallop striated muscle (34) (1SR6) or squid myosin in the Mg.ADP state (3I5F) are docked onto that of the

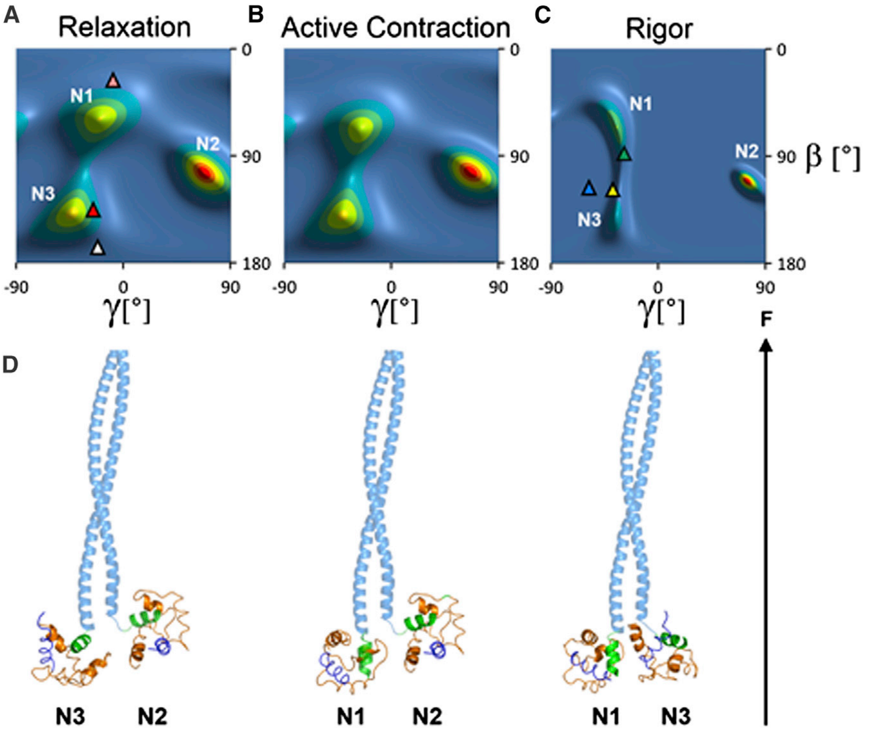

FIGURE 3 Orientation of the RLC N-lobe in cardiac trabeculae. (*A–C*) Orientation distributions of the RLC N-lobe ($(\beta,\gamma)_{DB}$) in relaxation, active isometric contraction, and rigor, respectively, calculated from the order parameters shown in Table S2 using RLC coordinates from PDB entry 1SR6. Triangles denote N-lobe orientations for the blocked (*red* (128°, −38°)) and free (*white* (163°, −24°)) heads of isolated thick filaments from invertebrate muscle (3DTP), and for the actin-bound rigor complex of chicken skeletal myosin S1 (2MYS; green (88°, −29°)), nucleotide-free scallop myosin (1SR6; *yellow* (120°, −45°)), smooth muscle myosin in the ADP.Pi state (1BR1; *pink* (24°, −6°)), and squid myosin in the Mg.ADP state (3I5F; *cyan* (120°, −59°)). (*D*) Structural models of pairs of N-lobes (1SR6 coordinates) in the N1, N2, or N3 orientations, with $(\beta,\gamma)_{DB}$ = (60°, −30°), (105°, 70°), and (135°, −45°), respectively, attached to part of the myosin subfragment 2 coiled-coil (human cardiac myosin S2Δ; PDB 2FXO). The myosin heavy chain is light blue (S2) and green; RLC is orange and its D-helix blue. The orientation of the filament axis (F) is indicated by the black arrow.

actin-bound 2MYS structure (Fig. 3 *C*, *yellow* and *cyan triangles*, respectively). The N3 peak is also close to the RLC N-lobe orientations of the blocked and free myosin heads of the dephosphorylated OFF or J-motif state of isolated invertebrate thick filaments (42) (Fig. 3 *A*, *red* and *white triangles*, respectively), a conformation that is also present in the C-zone of thick filaments from cardiac muscle, the region that contains MyBP-C (43,44). The N2 peak does not correspond to any N-lobe orientation reported previously.

Orientation of the cRLC C-lobe

The orientation of the C-lobe of the cRLC was described in terms of its local EG helix frame $(\beta,\gamma)_{EG}$. ME orientation maps of the RLC C-lobe in this frame showed four peaks in relaxation (Fig. 4 *A*) that we refer to as C1 to C4 in order of increasing β, with approximate peak coordinates $(\beta,\gamma)_{DB}$ = (30°, 15°), (70°, −60°), (90°, 30°), and (125°, −25°), respectively. Peaks C1 and C3 were weaker during active contraction (Fig. 4 *B*) than in relaxation, and no longer appear as distinct peaks in rigor (Fig. 4 *C*). Peak C4 was more intense than C2 in rigor, a reversal of their relative intensities in relaxation and active contraction.

The orientation of the RLC C-lobe in the chicken skeletal myosin head in the nucleotide-free state (2MYS) bound to the actin filament in rigor (Fig. 4 *C*, *green diamond*) is close to the C4 peak. The C-lobe orientation of the actin-bound scallop myosin structure (1SR6), calculated as described previously (*yellow diamond*) is even closer to C4, but that of the squid myosin head in the ADP state (3I5F; *cyan diamond*) has a very different γ, effectively bringing it close to C2 when the symmetry-generated wrapping of the maps is taken into account. The orientations of the RLC C-lobes in the free and blocked heads in isolated thick filaments

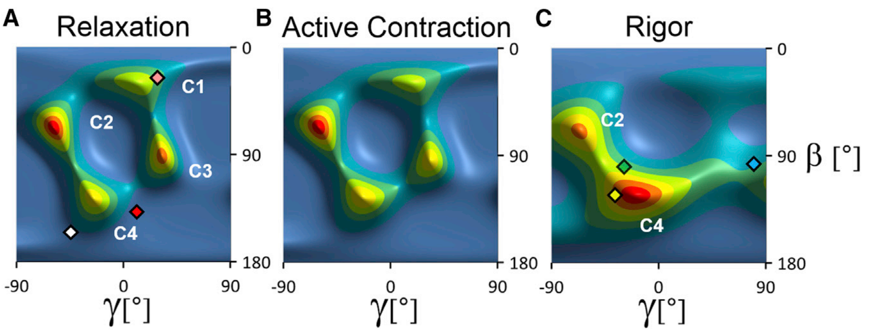

FIGURE 4 Orientation of the RLC C-lobe in cardiac trabeculae. Orientation distributions of the RLC C-lobe ($(\beta,\gamma)_{EG}$) in relaxation, active isometric contraction, and rigor, respectively, calculated from the order parameters shown in Table S2 using RLC coordinates from PDB entry 1SR6. Diamonds denote C-lobe orientations for the blocked (*red* (131°, 0°)) and free (*white* (158°, −60°)) heads of isolated thick filaments from invertebrate muscle (3DTP), and for the actin-bound rigor complex of chicken skeletal myosin (2MYS; *green* (100°, −28°)), scallop myosin (1SR6; *yellow* (125°, −35°)), smooth muscle myosin in the ADP.Pi state (1BR1; *pink* (31°, 20°)), and squid myosin in the Mg.ADP state (3I5F; *cyan* (95°, 82°)).

from invertebrate muscle (Fig. 4 *A*, *white* and *red diamonds*), are closest to C4. The actin-bound pre-powerstroke state modeled using the smooth muscle myosin head structure in the ADP.Pi state (1BR1) as described above (Fig. 4 *A*, *pink diamond*) superimposes on C1. The C3 peak does not correspond to any C-lobe orientation reported previously.

Although our data do not allow a unique assignment of the N-lobe peaks (Fig. 3) to C-lobe peaks (Fig. 4), the conserved fold of the RLC seen in crystal structures (Fig. S3) indicates that, if the RLC has a similar fold in trabeculae, its D- and E-helices are expected to be almost parallel, and therefore for a given RLC conformation $(\beta)_{DB}$ for the N lobe (Fig. 3) is approximately equal to $(\beta)_{EG}$ for the C lobe (Fig. 4). The DB and EG reference frames have also been chosen so that $(\gamma)_{DB}$ is approximately equal to $(\gamma)_{EG}$. Thus, it seems likely that the N2 and N3 peaks (Fig. 3) correspond to the C-lobe peaks C3 and C4 (Fig. 4), respectively, and N1 may correspond to a fusion of C1 and C2.

DISCUSSION

Determination of cRLC lobe orientations in cardiac muscle by polarized fluorescence

The orientations of the N- and C-lobes of the cRLC in ventricular trabeculae were determined by attaching a BSR probe to one of four sites in each lobe, replacing a small fraction of the native cRLC by BSR-cRLC, and using polarized fluorescence to obtain information about the in situ orientation of each BSR probe. The data from the four probes in each lobe were then combined using a maximum entropy algorithm and crystallographic data on the relative orientation of the probes in the local reference frame of the protein to calculate the distribution of the orientations of each lobe with respect to the trabecular or filament axis. The maximum entropy approach gives an exact but not unique fit to the measured order parameters; it provides the smoothest orientation distribution consistent with those parameters and with the angular relationships between the probes in the reference frame. Although features with very high orientational resolution may not be accurately characterized, the method successfully recovers multiple orientation populations when these are separated by angles of tens of degrees (33), as in the distributions reported here.

The dependence of the positions, shapes, separation, and relative intensities of the peaks in these orientation distributions on the choice of RLC reference structure (Fig. S4) showed that the variations of the fold of the RLC backbone between these structures (Fig. S3), which are from myosins from a very wide range of muscle types and species, are sufficiently large to shift the peaks by up to 30° in extreme cases for the N-lobe, with smaller shifts, typically 5–10°, for the C-lobe. These orientation distributions should therefore be regarded as approximate or relatively low-resolution representations of the real cRLC lobe orientations in trabeculae, with an uncertainty of 10–20° in the peak orientations related to the uncertainty in the in situ fold of each cRLC lobe. In addition, although the precision of the measured order parameters is sufficient to constrain the peak orientations at this angular resolution, the relative areas of the peaks do not give a precise measure of the angular disorder or the numbers of myosin molecules in each peak (see the Supporting Material and Fig. S6 and Fig.S7). Finally, as in any determination of orientation using dipole probes in the quasicylindrical symmetry of muscle cells, it is not possible to distinguish between up and down orientations with respect to the filament or trabecular axis, i.e., between (β, γ) and $(180°-\beta, 180°+\gamma)$, or to determine the azimuthal orientation of the lobe around the filament.

Comparison of RLC lobe orientations in cardiac and skeletal muscle

The N- and C-lobes of cRLC have multiple preferred orientations in heart muscle, with three peaks in the orientation distributions for the N-lobe (Fig. 3) and four for the C-lobe (Fig. 4). These orientation distributions were compared with those reported previously for the two lobes of the RLC in skeletal muscle (21–24) by transforming the skeletal muscle data into the DB and EG coordinate frames used here (Fig. S5). The peak of the N-lobe orientation distribution in relaxed skeletal muscle ((23,24) is approximately $(\beta,\gamma)_{DB} = (85°, -55°)$ (Fig. S5 *A*), which lies between the N1 and N3 peaks and the symmetry-related equivalent of the N2 peak in relaxed cardiac muscle (Fig. S5 *C*, Fig. 3 *A*). The C-lobe orientation distribution in relaxed skeletal muscle (23) has a peak near $(\beta,\gamma)_{EG} = (70°, -45°)$ (Fig. S5 *B*), close to the C2 peak in cardiac muscle (Fig. S5 *D*, Fig. 4 *A*), with a broad shoulder in the region of C3 and C4. Thus, the mean orientation of each RLC lobe is approximately the same in the two muscle types, but the orientation distributions in relaxed skeletal muscle are broader and simpler, which may be related to the use of smooth muscle RLC isoforms and/or more extreme RLC exchange conditions in the earlier skeletal muscle studies. Consistent with this explanation, a recent study of RLC orientation in skeletal muscle fibers using skeletal muscle RLC isoforms and a RLC exchange protocol similar to that used here showed multiple orientation peaks (37) (L. Fusi, King's College London, 2014, personal communication). Moreover, in contrast with the older studies of skeletal muscle RLC orientation (23), the temperature-dependent ordering of thick filament structure in relaxed muscle (45) was preserved in the recent study (37). These results indicate that the use of milder RLC exchange conditions and matched RLC isoform, as in this work, better preserves the native RLC orientation distributions. The

presence of multiple populations of myosin heads with distinct orientations in these distributions is likely to be related to the heterogeneity of nucleotide- and actin-binding states of the heads and the complexity of head-head and head-filament interactions in the intact filament lattice.

Comparison of RLC lobe orientations in cardiac muscle with those in isolated filaments

Electron micrographs of isolated thick filaments from invertebrate muscle in the dephosphorylated OFF state show pairs of myosin heads folded back against the filament backbone (42) in a J-motif (Fig. 5 *A*), first described for vertebrate smooth muscle myosin (46). A similar conformation has been seen in electron micrographs of the C-zone of isolated thick filaments from mammalian cardiac muscle, the region containing MyBP-C (43,44), and in electron tomograms of relaxed skeletal muscle (47). The two myosin heads in the J-motif are nonequivalent, and are conventionally described as blocked and free heads, with distinct orientations of both the catalytic and light chain domains (Fig. 5 *A*). The limited resolution of the electron micrographs did not allow the orientation of the RLC lobes in this structure to be determined directly, but the regions of the myosin heavy chain that bind the RLC (*darker green* in Fig. 5) were fitted to the electron density by allowing flexibility between the ELC and RLC regions (*red arrowheads*) and between the catalytic and light chain domains (*blue arrowheads*). The J-motif state is considered to be a model for the relaxed or diastolic structure of the myosin heads in cardiac thick filaments, and our data allow this idea to be tested. The approximate correspondence between the RLC lobe orientations calculated from the J-motif model (Fig. 5 *A*) and the N3 (Fig. 3 *A*) and C4 (Fig. 4 *A*) peaks suggests that there is a population of myosin heads in the J-motif conformation in relaxed cardiac trabeculae, although other heads are clearly not in the J-motif conformation. Moreover the latter conformation is not confined to the relaxed state, because similar features are observed during active contraction (Fig. 3 *B* and Fig. 4 *B*).

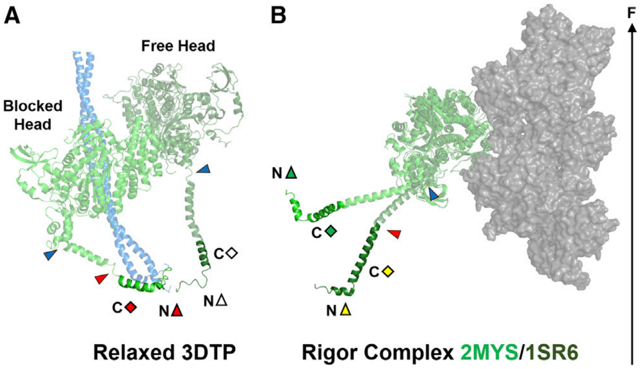

FIGURE 5 Orientation of myosin head domains in relaxation and rigor. (*A*) Blocked and free myosin heads (*green*) in the J-motif of invertebrate thick filaments (PDB 3DTP (42,62)) folded back against the S2 coiled-coil (*light blue*). (*B*) Myosin heads bound to actin (*gray*) in the rigor complex for the 2MYS (41) (*upper*) and 1SR6 (34) (*lower*) conformations. The triangles and diamonds correspond to the symbols in Figs. 3 and 4, respectively. Potential hinges between the catalytic and light chain domain and between the RLC and ELC regions are indicated by blue and red arrowheads, respectively. Orientations are shown with respect to a vertical filament axis (*black arrow*). The myosin light chains have been omitted for clarity.

The orientation of the RLC region of myosin heads in the rigor state has also been estimated previously by fitting crystal structures of the myosin head domain into electron micrographs of isolated actin filaments decorated with proteolytic myosin head fragments in the absence of ATP. When this procedure is carried out using the crystal structure of nucleotide-free chicken skeletal muscle myosin heads (2MYS) (41) (Fig. 5 *B*, upper conformation of the myosin heavy chain; *green*), the calculated RLC N-lobe orientation in the DB frame is close to N1 (Fig. 3 *C*, *green triangle*), and the C-lobe EG frame orientation is close to C4 (Fig. 4 *C*, *green diamond*). This result depends on which crystal structure is used to model the myosin head conformation. When the catalytic domain of scallop striated muscle myosin in the nucleotide-free state (34) (1SR6) is docked onto that of the rigor actin-myosin head structure of 2MYS (41) the light chain domain is much more parallel to the filament axis (Fig. 5 *B*, *lower* myosin heavy chain conformation), the N-lobe orientation is closer to the N3 peak (Fig. 3 *C*, *yellow triangle*), and the C-lobe orientation is even closer to C4 (Fig. 4 *C*, *yellow diamond*). Thus, the vertical density joining peaks N1 and N3 in the rigor orientation distribution (Fig. 3 *C*) corresponds to tilting of the light chain domain in the plane corresponding to the major bend observed between the 2MYS and 1SR6 crystal structures when their catalytic domains are bound to actin in rigor conditions, although this tilting is somewhat magnified in situ. The actin-bound pre-powerstroke state, modeled using the smooth muscle myosin head ADP.Pi structure (1BR1) (Fig. 3 *A*, *pink triangle*, Fig. 4 *A*, *pink diamond*), is close to N1 and C1, suggesting that these pre-powerstroke myosin head conformations may also be present during relaxation and active contraction. However, the great diversity of bends in the light chain regions of crystal structures of myosin heads from different species, especially in the twist angle γ as exemplified by the differences between C-lobe conformations of actin-bound chicken, scallop, and squid myosins (Fig. 4 *C*, *diamonds*), suggests that these apparent correlations between in situ and crystallographic structures should be treated with caution.

The similarity of the RLC N-lobe orientation in the relaxed J-motif (Fig. 5 *A*) and the rigor actin-myosin complex (Fig. 5 *B*) appears not to have been noted previously, but has a potentially fundamental functional consequence. It implies that a myosin head could make the transition from relaxed J-motif packing on the surface of the thick filament to strong binding to actin in rigor with little change in the orientation of its RLC N-lobe, although a large change in

the orientation of the catalytic domain would of course be required.

Implications for the function of the RLC region of myosin in cardiac muscle

In the context of conventional models of muscle contraction and its regulation, in which the myosin heads are generally thought to be close to the thick filament surface in relaxation but released from the thick filament surface and bound to the thin filaments in active contraction, the broad similarity of the orientation distributions of the RLC N- and C-lobes in cardiac muscle in these two states, a similarity that extends to rigor for the N-lobe (Figs. 3 and 4) is unexpected. For the N3 and C4 orientations, this similarity might be related to the similar orientation of the RLC region in the relaxed J-motif and actin-bound rigor states discussed previously, which suggests that myosin heads with their RLC regions in the J-motif conformation are able to bind actin without changing that conformation, presumably by a large-scale change in the conformation of the rest of the head. However, the observation that the other N-lobe and C-lobe peaks are also largely preserved during activation suggests that the significant fraction of heads that are not in the J-motif in relaxed muscle also maintain their RLC orientation on calcium activation.

Thus, the transition between the relaxed state, in which nearly all myosin heads are expected to be detached from actin, and isometric contraction, in which a substantial fraction of the mass of the heads has moved toward the thin filaments in both skeletal and cardiac muscle (48,49), is accompanied by little change in the overall orientation distribution of either the N- or the C-lobe of cRLC (Figs. 3 and 4). These results suggest that the organization of the RLC region of the myosin heads on the surface of the thick filaments is largely insensitive to calcium activation, and that the major component of the motion of the myosin heads associated with muscle activation occurs by bending in the heads between the RLC region and the catalytic domain. The almost constant orientation of the RLC regions during activation is likely to be due to a continuing interaction with the surface of the thick filament, presumably through the N-lobe, because the C-lobe orientation changes significantly in rigor (Fig. 4 *C*). The maintenance of a large population of myosin heads in a conformation characteristic of relaxed muscle during calcium activation of cardiac muscle is consistent with the presence of a substantial fraction of the heads with very slow nucleotide turnover in these conditions (50).

The above conclusions imply a significant modification of cross-bridge models in which the long S2 connection between the myosin head and the filament backbone (Fig. 1) provides the radial and azimuthal flexibility to allow myosin heads to attach optimally to actin sites in the three-dimensional lattice of thin and thick filaments in the muscle sarcomere (1). However the required radial flexibility is small; in mouse hearts in vivo, the interfilament spacing is ~5% larger at systole than at diastole (51), corresponding to an increase in the center-to-center distance between thin and thick filaments of only 1.3 nm. Some azimuthal flexibility might also be retained in the presence of RLC/S2/filament backbone interactions if these are electrostatic rather than stereospecific (52).

Another implication of the conclusion that the RLC remains docked on the thick filament surface during active contraction would be a modification of the tilting lever arm model for the action of the myosin motor (5,41,53). In the generally accepted form of this model, force and filament sliding in muscle are generated by tilting of the LCD of myosin, whereas the catalytic domain remains docked in a fixed conformation on actin. Our results suggest that, in situ, the functional lever arm might be composed of only part of the LCD, and that the effective pivot between the myosin head and the thick-filament associated portion of each myosin molecule may be between the N- and C-lobes of the RLC or between the RLC and the ELC, rather than at the S1-S2 junction as usually assumed. The difference between RLC C-lobe orientations in active contraction and rigor (Fig. 4) suggests that the effective pivot is likely to be between the N- and C-lobes of the RLC. Bifunctional rhodamine probes on the C-lobe of the RLC rotate both during the elastic response and during the working stroke of the actin-attached head (6,21,22), consistent with this location of the pivot, which would shorten the lever arm from the canonical 11 nm to ~9 nm.

Finally, although our results show that the orientation of the RLC region of the myosin head on the surface of the thick filament is insensitive to the increase in free calcium concentration associated with muscle activation, it remains possible that the RLC region could exert longer term control on the conformation of the myosin heads, and thus on cardiac contractility. The orientation distributions reported here relate to the fully dephosphorylated state of the RLC, but the N-lobe of cRLC can be phosphorylated by cardiac myosin light chain kinase (54,55) and may interact with unphosphorylated cMyBP-C (19). Both of these interactions can alter myosin head orientation (56–58). Thus, our conclusion that the cRLC acts as a thick filament binding domain in the dephosphorylated state suggests a general structural mechanism by which modification of the cRLC could control cardiac contractility, which might also be tested by future studies using this technique.

CONCLUSIONS

In heart muscle cells, the orientation distribution of the N-lobe of the myosin regulatory light chain is broadly similar in relaxation, isometric contraction, and in rigor, and that of its C-lobe is similar in relaxation and active contraction. These results suggest that the conformational changes of

the myosin heads associated with activation of heart muscle take place while their RLC regions remain bound to the thick filament. In cardiac muscle, the orientation of the RLC region may be primarily determined by its interaction with the thick filament, and the lever arm of the myosin motor may be shorter than previously thought.

SUPPORTING MATERIAL

Supporting Materials and Methods, seven figures, and three tables are available at http://www.biophysj.org/biophysj/supplemental/S0006-3495(14)01248-X.

ACKNOWLEDGMENTS

The authors thank Tam T. Bui and Alex Drake (King's College London, Biomolecular Spectroscopy Centre, Guy's Campus, London SE1 1UL) for their expertise and help with CD Spectroscopy. We are grateful to Mathias Gautel, David R. Trentham, Andrea Knowles, and Birgit Brandmeier (Randall Division of Cell and Molecular Biophysics, King's College London, SE1 1UL) for help and advice and to the British Heart Foundation for financial support.

SUPPORTING CITATIONS

References (59,60) appear in the Supporting Material.

REFERENCES

1. Huxley, H. E. 1969. The mechanism of muscular contraction. *Science.* 164:1356–1365.
2. Huxley, A. F., and R. M. Simmons. 1971. Proposed mechanism of force generation in striated muscle. *Nature.* 233:533–538.
3. Lymn, R. W., and E. W. Taylor. 1971. Mechanism of adenosine triphosphate hydrolysis by actomyosin. *Biochemistry.* 10:4617–4624.
4. Dominguez, R., Y. Freyzon, …, C. Cohen. 1998. Crystal structure of a vertebrate smooth muscle myosin motor domain and its complex with the essential light chain: visualization of the pre-power stroke state. *Cell.* 94:559–571.
5. Rayment, I., W. R. Rypniewski, …, H. M. Holden. 1993. Three-dimensional structure of myosin subfragment-1: a molecular motor. *Science.* 261:50–58.
6. Irving, M., T. St Claire Allen, …, Y. E. Goldman. 1995. Tilting of the light-chain region of myosin during step length changes and active force generation in skeletal muscle. *Nature.* 375:688–691.
7. Holmes, K. C. 1997. The swinging lever-arm hypothesis of muscle contraction. *Curr. Biol.* 7:R112–R118.
8. Houdusse, A., A. G. Szent-Gyorgyi, and C. Cohen. 2000. Three conformational states of scallop myosin S1. *Proc. Natl. Acad. Sci. USA.* 97:11238–11243.
9. Brown, J. H., V. S. Kumar, …, C. Cohen. 2011. Visualizing key hinges and a potential major source of compliance in the lever arm of myosin. *Proc. Natl. Acad. Sci. USA.* 108:114–119.
10. Gourinath, S., D. M. Himmel, …, C. Cohen. 2003. Crystal structure of scallop Myosin S1 in the pre-power stroke state to 2.6 Å resolution: flexibility and function in the head. *Structure.* 11:1621–1627.
11. Elliott, A., and G. Offer. 1978. Shape and flexibility of the myosin molecule. *J. Mol. Biol.* 123:505–519.
12. Gordon, A. M., E. Homsher, and M. Regnier. 2000. Regulation of contraction in striated muscle. *Physiol. Rev.* 80:853–924.
13. Tobacman, L. S. 1996. Thin filament-mediated regulation of cardiac contraction. *Annu. Rev. Physiol.* 58:447–481.
14. Colson, B. A., S. J. Gruber, and D. D. Thomas. 2012. Structural dynamics of muscle protein phosphorylation. *J. Muscle Res. Cell Motil.* 33:419–429.
15. Scruggs, S. B., and R. J. Solaro. 2011. The significance of regulatory light chain phosphorylation in cardiac physiology. *Arch. Biochem. Biophys.* 510:129–134.
16. Pfuhl, M., and M. Gautel. 2012. Structure, interactions and function of the N-terminus of cardiac myosin binding protein C (MyBP-C): who does what, with what, and to whom? *J. Muscle Res. Cell Motil.* 33:83–94.
17. Flashman, E., C. Redwood, …, H. Watkins. 2004. Cardiac myosin binding protein C: its role in physiology and disease. *Circ. Res.* 94:1279–1289.
18. Scruggs, S. B., A. C. Hinken, …, R. J. Solaro. 2009. Ablation of ventricular myosin regulatory light chain phosphorylation in mice causes cardiac dysfunction in situ and affects neighboring myofilament protein phosphorylation. *J. Biol. Chem.* 284:5097–5106.
19. Ratti, J., E. Rostkova, …, M. Pfuhl. 2011. Structure and interactions of myosin-binding protein C domain C0: cardiac-specific regulation of myosin at its neck? *J. Biol. Chem.* 286:12650–12658.
20. Szczesna-Cordary, D., G. Guzman, …, J. Zhao. 2004. Familial hypertrophic cardiomyopathy-linked alterations in Ca2+ binding of human cardiac myosin regulatory light chain affect cardiac muscle contraction. *J. Biol. Chem.* 279:3535–3542.
21. Corrie, J. E., B. D. Brandmeier, …, M. Irving. 1999. Dynamic measurement of myosin light-chain-domain tilt and twist in muscle contraction. *Nature.* 400:425–430.
22. Hopkins, S. C., C. Sabido-David, …, Y. E. Goldman. 2002. Orientation changes of the myosin light chain domain during filament sliding in active and rigor muscle. *J. Mol. Biol.* 318:1275–1291.
23. Brack, A. S., B. D. Brandmeier, …, M. Irving. 2004. Bifunctional rhodamine probes of Myosin regulatory light chain orientation in relaxed skeletal muscle fibers. *Biophys. J.* 86:2329–2341.
24. Romano, D., B. D. Brandmeier, …, M. Irving. 2012. Orientation of the N-terminal lobe of the myosin regulatory light chain in skeletal muscle fibers. *Biophys. J.* 102:1418–1426.
25. De Simone, A., J. E. Corrie, …, F. Fraternali. 2008. Conformation and dynamics of a rhodamine probe attached at two sites on a protein: implications for molecular structure determination in situ. *J. Am. Chem. Soc.* 130:17120–17128.
26. Laemmli, U. K. 1970. Cleavage of structural proteins during the assembly of the head of bacteriophage T4. *Nature.* 227:680–685.
27. Kinoshita, E., E. Kinoshita-Kikuta, …, T. Koike. 2006. Phosphate-binding tag, a new tool to visualize phosphorylated proteins. *Mol. Cell. Proteomics.* 5:749–757.
28. Ling, N., C. Shrimpton, …, M. Irving. 1996. Fluorescent probes of the orientation of myosin regulatory light chains in relaxed, rigor, and contracting muscle. *Biophys. J.* 70:1836–1846.
29. Hopkins, S. C., C. Sabido-David, …, Y. E. Goldman. 1998. Fluorescence polarization transients from rhodamine isomers on the myosin regulatory light chain in skeletal muscle fibers. *Biophys. J.* 74:3093–3110.
30. Sun, Y. B., F. Lou, and M. Irving. 2009. Calcium- and myosin-dependent changes in troponin structure during activation of heart muscle. *J. Physiol.* 587:155–163.
31. Dale, R. E., S. C. Hopkins, …, Y. E. Goldman. 1999. Model-independent analysis of the orientation of fluorescent probes with restricted mobility in muscle fibers. *Biophys. J.* 76:1606–1618.
32. Julien, O., Y. B. Sun, …, M. Irving. 2007. Toward protein structure in situ: comparison of two bifunctional rhodamine adducts of troponin C. *Biophys. J.* 93:1008–1020.
33. van der Heide, U. A., S. C. Hopkins, and Y. E. Goldman. 2000. A maximum entropy analysis of protein orientations using fluorescence polarization data from multiple probes. *Biophys. J.* 78:2138–2150.
34. Risal, D., S. Gourinath, …, C. Cohen. 2004. Myosin subfragment 1 structures reveal a partially bound nucleotide and a complex salt bridge

that helps couple nucleotide and actin binding. *Proc. Natl. Acad. Sci. USA*. 101:8930–8935.

35. Holzwarth, G., and P. Doty. 1965. The ultraviolet circular dichroism of polypeptides. *J. Am. Chem. Soc.* 87:218–228.

36. Szczesna, D., D. Ghosh, ..., J. D. Potter. 2001. Familial hypertrophic cardiomyopathy mutations in the regulatory light chains of myosin affect their structure, Ca2+ binding, and phosphorylation. *J. Biol. Chem.* 276:7086–7092.

37. Fusi, L., Z. Huang, and M. Irving. 2014. Changes in the orientation of the myosin light chain domain (LCD) associated with thick filament-based regulation of skeletal muscle. *Biophys. J.* 106 (*Suppl 1*): 724a–725a.

38. Walker, L. A., A. M. Medway, ..., P. M. Buttrick. 2011. Tissue procurement strategies affect the protein biochemistry of human heart samples. *J. Muscle Res. Cell Motil.* 31:309–314.

39. Wilson, I. B., and B. Ginsburg. 1955. A powerful reactivator of alkylphosphate-inhibited acetylcholinesterase. *Biochim. Biophys. Acta.* 18:168–170.

40. Olsson, M. C., J. R. Patel, ..., R. L. Moss. 2004. Basal myosin light chain phosphorylation is a determinant of Ca2+ sensitivity of force and activation dependence of the kinetics of myocardial force development. *Am. J. Physiol. Heart Circ. Physiol.* 287:H2712–H2718.

41. Rayment, I., H. M. Holden, ..., R. A. Milligan. 1993. Structure of the actin-myosin complex and its implications for muscle contraction. *Science.* 261:58–65.

42. Woodhead, J. L., F. Q. Zhao, ..., R. Padrón. 2005. Atomic model of a myosin filament in the relaxed state. *Nature.* 436:1195–1199.

43. Al-Khayat, H. A., R. W. Kensler, ..., E. P. Morris. 2013. Atomic model of the human cardiac muscle myosin filament. *Proc. Natl. Acad. Sci. USA*. 110:318–323.

44. Zoghbi, M. E., J. L. Woodhead, ..., R. Craig. 2008. Three-dimensional structure of vertebrate cardiac muscle myosin filaments. *Proc. Natl. Acad. Sci. USA*. 105:2386–2390.

45. Lowy, J., D. Popp, and A. A. Stewart. 1991. X-ray studies of order-disorder transitions in the myosin heads of skinned rabbit psoas muscles. *Biophys. J.* 60:812–824.

46. Wendt, T., D. Taylor, ..., K. Taylor. 2001. Three-dimensional image reconstruction of dephosphorylated smooth muscle heavy meromyosin reveals asymmetry in the interaction between myosin heads and placement of subfragment 2. *Proc. Natl. Acad. Sci. USA*. 98:4361–4366.

47. Luther, P. K., H. Winkler, ..., J. Liu. 2011. Direct visualization of myosin-binding protein C bridging myosin and actin filaments in intact muscle. *Proc. Natl. Acad. Sci. USA*. 108:11423–11428.

48. Haselgrove, J. C., and H. E. Huxley. 1973. X-ray evidence for radial cross-bridge movement and for the sliding filament model in actively contracting skeletal muscle. *J. Mol. Biol.* 77:549–568.

49. Matsubara, I., N. Yagi, and M. Endoh. 1979. Movement of myosin heads during a heart beat. *Nature.* 278:474–476.

50. Hooijman, P., M. A. Stewart, and R. Cooke. 2011. A new state of cardiac myosin with very slow ATP turnover: a potential cardioprotective mechanism in the heart. *Biophys. J.* 100:1969–1976.

51. Toh, R., M. Shinohara, ..., N. Yagi. 2006. An X-Ray diffraction study on mouse cardiac cross-bridge function in vivo: effects of adrenergic beta-stimulation. *Biophys. J.* 90:1723–1728.

52. Blankenfeldt, W., N. H. Thomä, ..., I. Schlichting. 2006. Crystal structures of human cardiac beta-myosin II S2-Delta provide insight into the functional role of the S2 subfragment. *Proc. Natl. Acad. Sci. USA*. 103:17713–17717.

53. Geeves, M. A., and K. C. Holmes. 1999. Structural mechanism of muscle contraction. *Annu. Rev. Biochem.* 68:687–728.

54. Chan, J. Y., M. Takeda, ..., H. Kasahara. 2008. Identification of cardiac-specific myosin light chain kinase. *Circ. Res.* 102:571–580.

55. Seguchi, O., S. Takashima, ..., M. Kitakaze. 2007. A cardiac myosin light chain kinase regulates sarcomere assembly in the vertebrate heart. *J. Clin. Invest.* 117:2812–2824.

56. Colson, B. A., M. R. Locher, ..., R. L. Moss. 2010. Differential roles of regulatory light chain and myosin binding protein-C phosphorylations in the modulation of cardiac force development. *J. Physiol.* 588:981–993.

57. Colson, B. A., T. Bekyarova, ..., R. L. Moss. 2007. Radial displacement of myosin cross-bridges in mouse myocardium due to ablation of myosin binding protein-C. *J. Mol. Biol.* 367:36–41.

58. Colson, B. A., T. Bekyarova, ..., R. L. Moss. 2008. Protein kinase A-mediated phosphorylation of cMyBP-C increases proximity of myosin heads to actin in resting myocardium. *Circ. Res.* 103:244–251.

59. Lawson, J. D., E. Pate, ..., R. G. Yount. 2004. Molecular dynamics analysis of structural factors influencing back door Pi release in myosin. *Biophys. J.* 86:3794–3803.

60. Debreczeni, J. E., L. Farkas, ..., L. Nyitray. 2005. Structural evidence for non-canonical binding of Ca2+ to a canonical EF-hand of a conventional myosin. *J. Biol. Chem.* 280:41458–41464.

61. Holmes, K. C., D. Popp, ..., W. Kabsch. 1990. Atomic model of the actin filament. *Nature.* 347:44–49.

62. Alamo, L., W. Wriggers, ..., R. Padrón. 2008. Three-dimensional reconstruction of tarantula myosin filaments suggests how phosphorylation may regulate myosin activity. *J. Mol. Biol.* 384:780–797.

Biophysical Journal

Supporting Material

Orientation of the N- and C-Terminal Lobes of the Myosin Regulatory Light Chain in Cardiac Muscle

Thomas Kampourakis, Yin-Biao Sun, and Malcolm Irving

Supplemental Text

Dependence of RLC orientation distributions on the choice of RLC reference structure

The *in situ* orientation distributions of the N- and C-lobes of the RLC shown in **Figs. 3** and **4** of the main text were calculated using maximum entropy (ME) analysis from the measured order parameters (**Table S2**) and the RLC structure of scallop striated muscle myosin with bound ADP (1SR6(1)). These ME calculations were repeated using the five other reference structures in **Fig. S3**. For the order parameters recorded for the N-lobe probes in relaxation and active isometric contraction, this procedure was successful for five of the six reference structures. For the remaining structure, from scallop smooth muscle myosin (3PN7(mol2) (2)), which has an internal bend in the D helix and a distinct β_{DB} value (**Fig. S3B**), no distribution of N-lobe orientations could reproduce the measured order parameters, suggesting that the *in situ* structure of cRLC in heart muscle in these conditions is different from that in the 3PN7(mol2) structure. In rigor conditions, three of the six crystallographic structures were inconsistent with the measured order parameters for the N-lobe probes, 3PN7(mol2), chicken skeletal myosin (2MYS (3)), and a scallop striated muscle myosin with bound ADP.VO_4 (1QVI (4)).

N-lobe orientation distributions calculated using the two reference structures in addition to 1SR6 that fitted the measured order parameters from the N-lobe probes in all conditions (**Fig. S4**) showed the same three peaks as the 1SR6-based analysis (**Fig. 3**), with somewhat different peak positions and relative amplitudes, as expected from the slightly different folds of the N-lobe (**Figs S3A** and **S3B**). The mean and SD of the peak $(\beta,\gamma)_{DB}$ positions of the N-lobe in relaxing conditions calculated from the five structures that fitted the measured order parameters were (50° ± 10° , -60° ± 30°), (75° ± 20° , 50° ± 20°) and (130° ± 30°, -40° ± 10°). Thus peak N3 is more variable in β, reflecting its tendency to partially merge with peak N1 for some *in vitro* structures. Peak N1, conversely, is more variable in γ, reflecting a tendency to merge with the (180°-β, 180°+γ) dipole-related equivalent of N2.

For the C-lobe probes, the maximum entropy analysis was successful for all six reference structures. In general the resulting orientation distributions were similar for the six structures, with mean ± SD $(\beta,\gamma)_{EG}$ values of (30° ± 5°, 20° ± 20°), (50° ± 10°, -60° ± 5°), (90° ± 5°, 35° ± 5°) and (125° ± 5°, -30° ± 5°) for peaks C1-C4. The smaller SDs compared with those reported above for the N-lobe probes reflect the fact that the structure of the C-lobe is more highly conserved than that of the N-lobe (**Fig. S3**). Example orientation distributions for relaxation, active isometric contraction, and rigor calculated using the 3PN7 and 2BL0 reference structures are shown in **Fig. S4**.

The effects of experimental errors in the measured order parameters on the 2D ME distributions

The variability in the ME maps was simulated by Monte Carlo analysis. The error distribution of each measured order parameter was modeled as a normal distribution using its experimental mean and SD. The resulting eight normal distributions for each experimental condition were randomly sampled to give a new set of eight $\langle P_2 \rangle$ and $\langle P_4 \rangle$ values that differ from the experimental means used for the original 2D ME calculations in a way that accurately reproduces the experimental variability in the $\langle P_2 \rangle$ and $\langle P_4 \rangle$ values in that condition. Ten example results of the application of this procedure are shown in Fig. S6 for the N lobe, and in Fig. S7 for the C lobe. These ten 2D ME distributions are qualitatively similar in each case, but in general there is greater variability in the amplitude and width of each peak than in the peak position. The panel at the lower right of Figs S6 and S7 shows the

SD of the ten distributions. To a first approximation these SD plots are scaled down versions of the original 2D ME maps shown in Figs 3 and 4 of the main paper, which were calculated from the mean value of each order parameter. Thus the errors in the 2D ME maps arising from the experimental variability of the measured order parameters are sufficiently small that they do not affect the conclusions of the present paper.

Supplemental Figures

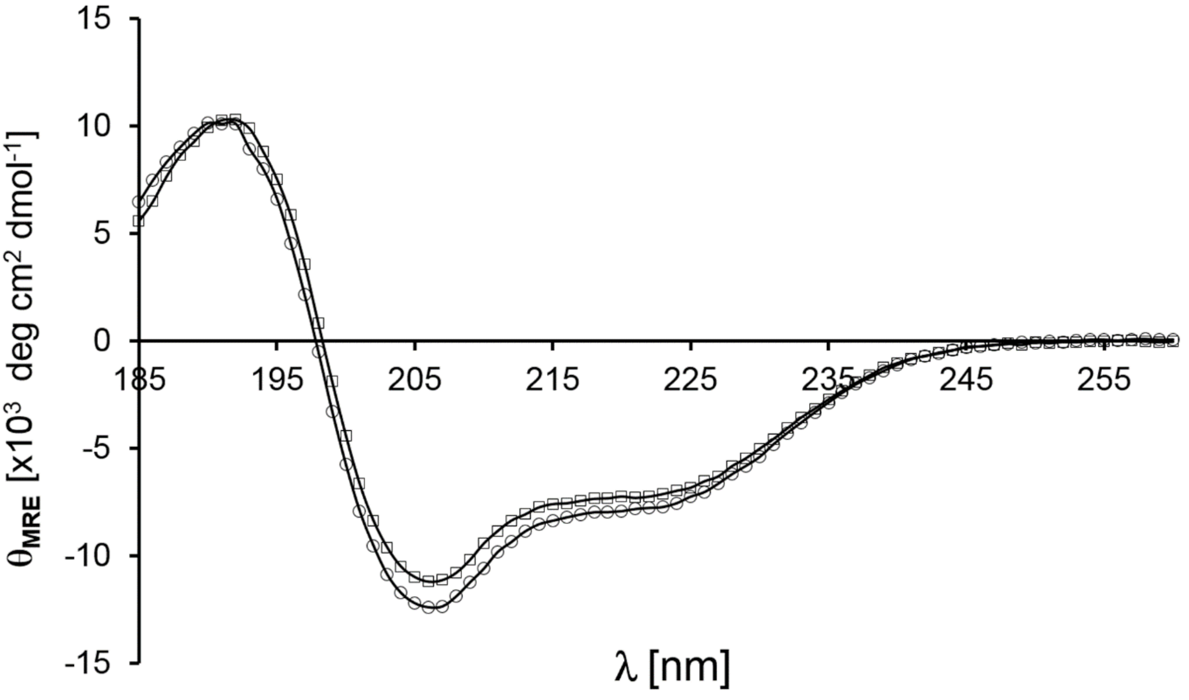

Figure S1. CD spectra of wild-type and BSR-labeled RLC.

Far-UV circular dichroism (CD) spectra of wild-type cRLC (squares with interpolated lines) and BSR-cRLC-G (circles with interpolated lines). Both spectra are typical for α-helical proteins with a high content of unfolded regions. The α-helical content in the BSR-cRLC-G is increased by 2% compared to wild-type, calculated using the standard equation for $[\theta]_{MRE}$ at 222 nm.

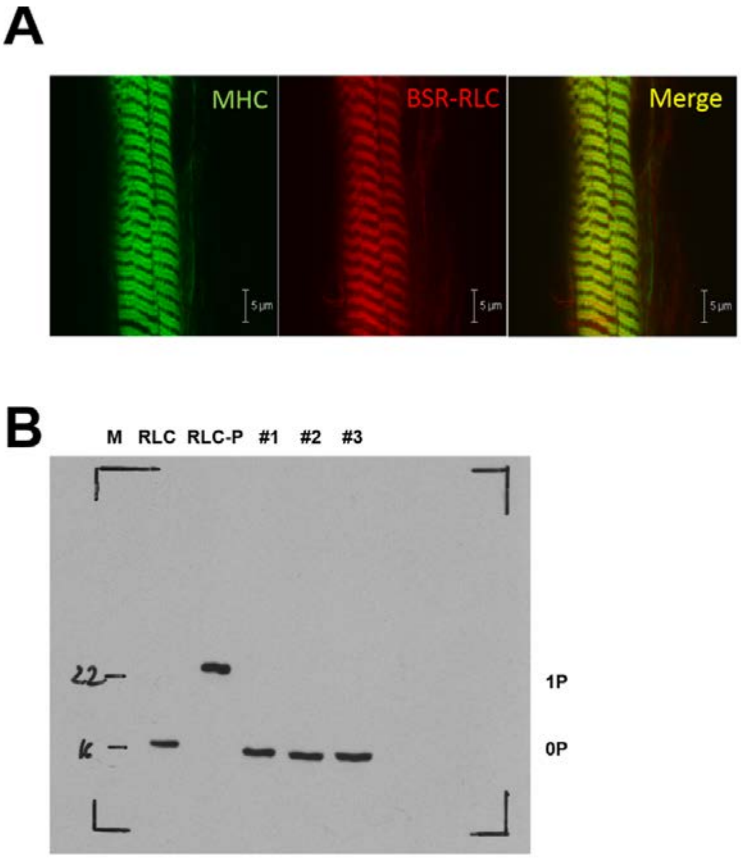

Figure S2. Sarcomeric localisation of BSR-cRLC and phosphorylation level of endogenous rat cRLC.

(**A**) Confocal microscopy image of a rat right ventricular trabecula after partial replacement of endogenous cRLC with BSR-cRLC-G (red channel) counterstained against myosin heavy chain (green). (**B**) The phosphorylation level of endogenous cRLCs from three different skinned rat trabecula preparations, determined by Phos-tag™ (5) SDS-PAGE followed by Western blot against cRLC (*RLC:* unphosphorylated recombinant cRLC; RLC-P: mono-phosphorylated recombinant cRLC; *#1=#3*: skinned trabeculae samples from three different hearts).

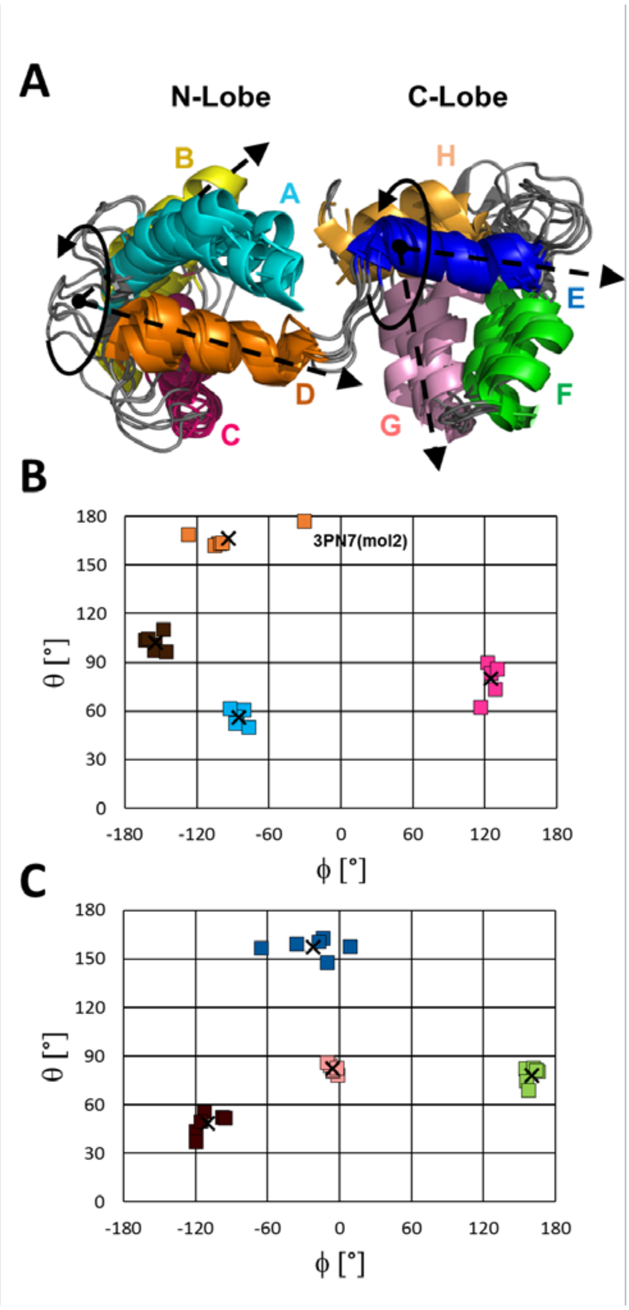

Figure S3. BSR dipole orientations in six reference structures of the RLC.

Dipole orientations (θ,ϕ) for BSR probes on the RLC N- and C-lobes calculated from different crystal structures (PDB entries 2MYS (3), 1SR6 (1), 3PN7 (2), 1QVI (6), 2BL0 (7)). (**A**) Structural alignment of RLC crystal structures: A-helix: cyan; B-helix: yellow; C-helix: magenta; D-helix: orange; E-helix: blue; F-helix: green; G-helix: pink; H-helix: light orange. The references axes describing the molecular references frames for the N- and C-lobe (DB- and EG-helix frame) are indicated by dashed arrows. (**B**) Probe dipole orientations on the N-lobe from different crystal structures with same colour coding as in (**A**); probe dipole of BSR crosslinking helices B and C shown in brown. (**C**) Probe dipole orientations on the C-lobe (FG-helix: brown). Black crosses indicate average probe dipole orientations for all structures.

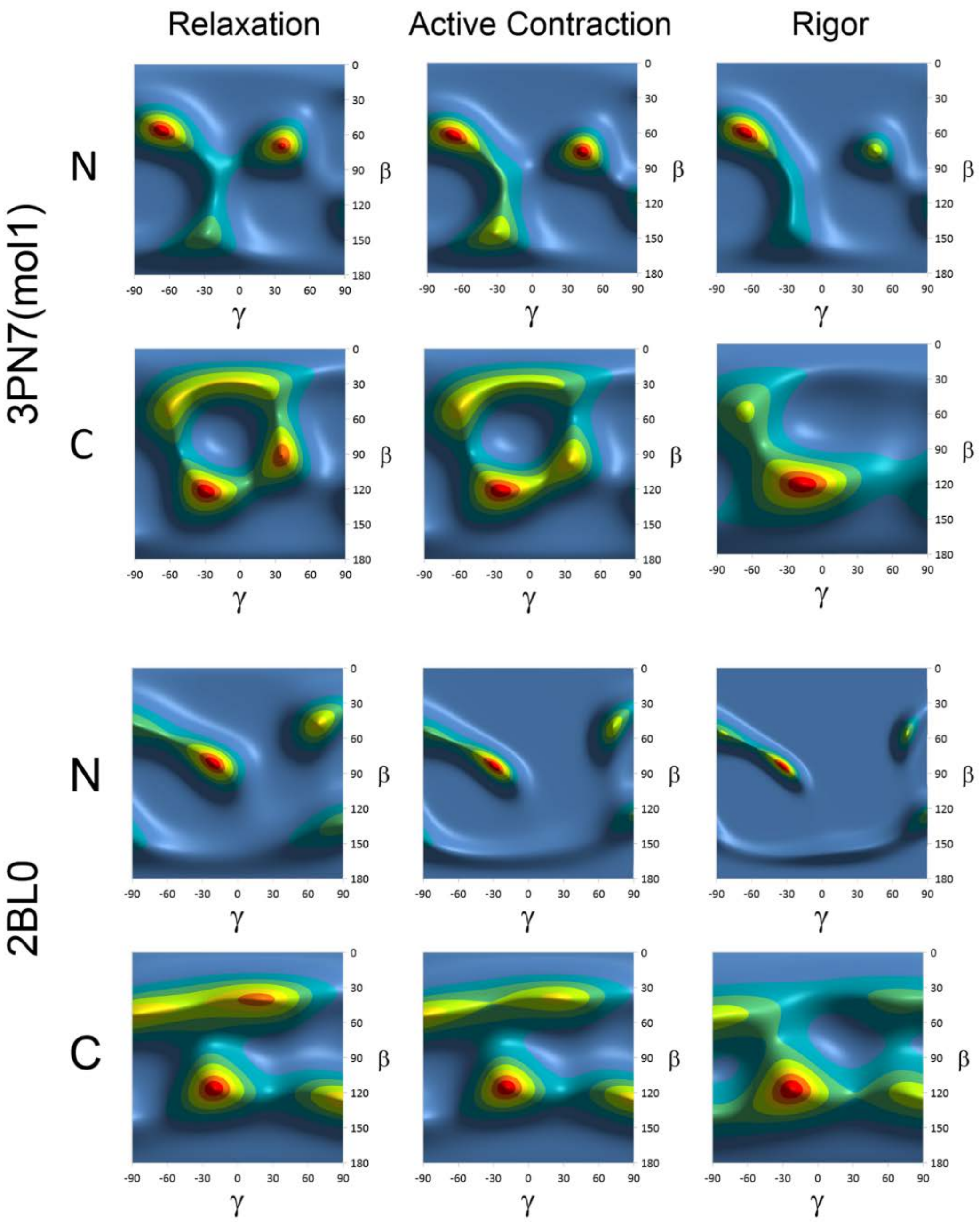

Figure S4. Maximum entropy distributions calculated using reference structures 3PN7(mol1) and 2BL0.

Maximum entropy distributions were calculated from order parameters for the N- and C-lobe RLC probes during relaxation, active isometric contraction and rigor (**Table S2**) using reference structures 3PN7(mol1) and 2BL0.

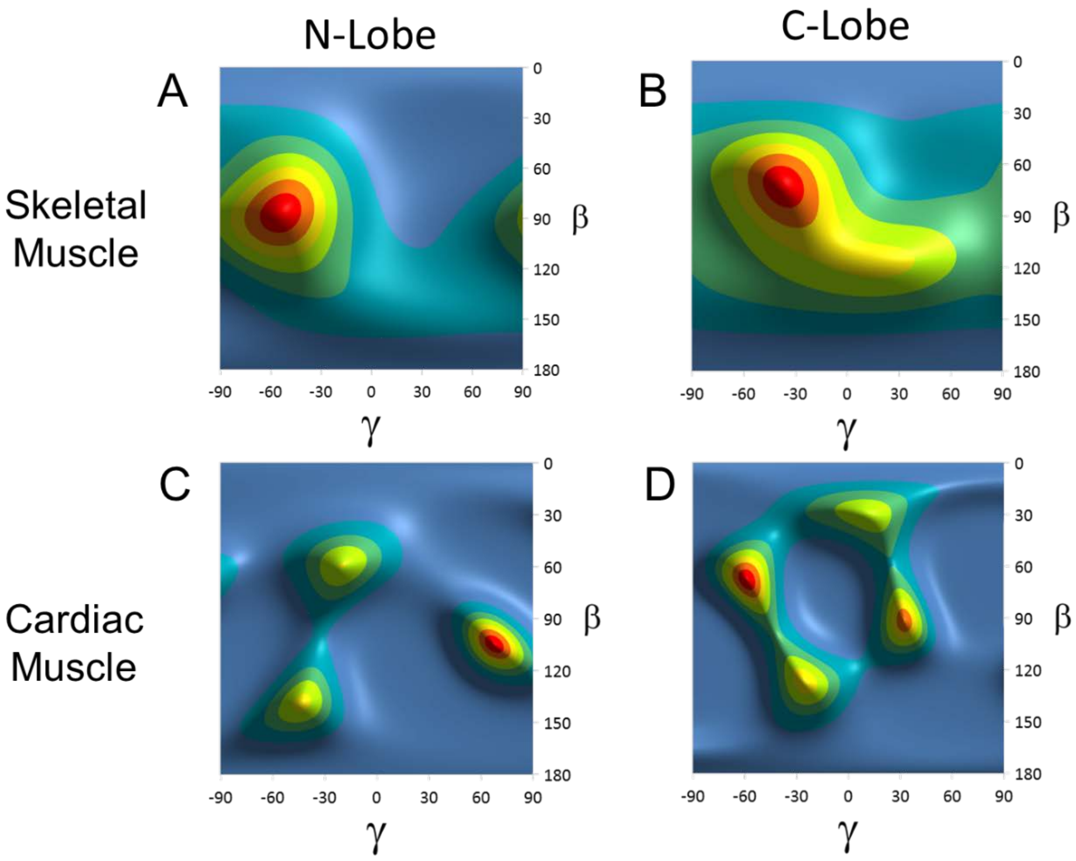

Figure S5. Comparison of orientation distributions of the N- and C-lobes of the RLC in relaxed skeletal and cardiac muscle.

(**A** and **B**). Maximum orientation distributions of the N- and C-lobes respectively of the RLC region of the myosin heads in relaxed skeletal muscle from the data of Romano et el ((8)) and Brack et al ((9)) transformed into the DB and EG coordinate frames used in the present work. (**C** and **D**). Maximum orientation distributions of the N- and C-lobes respectively of the RLC in relaxed cardiac muscle from Figs. 3 and 4 of the main text.

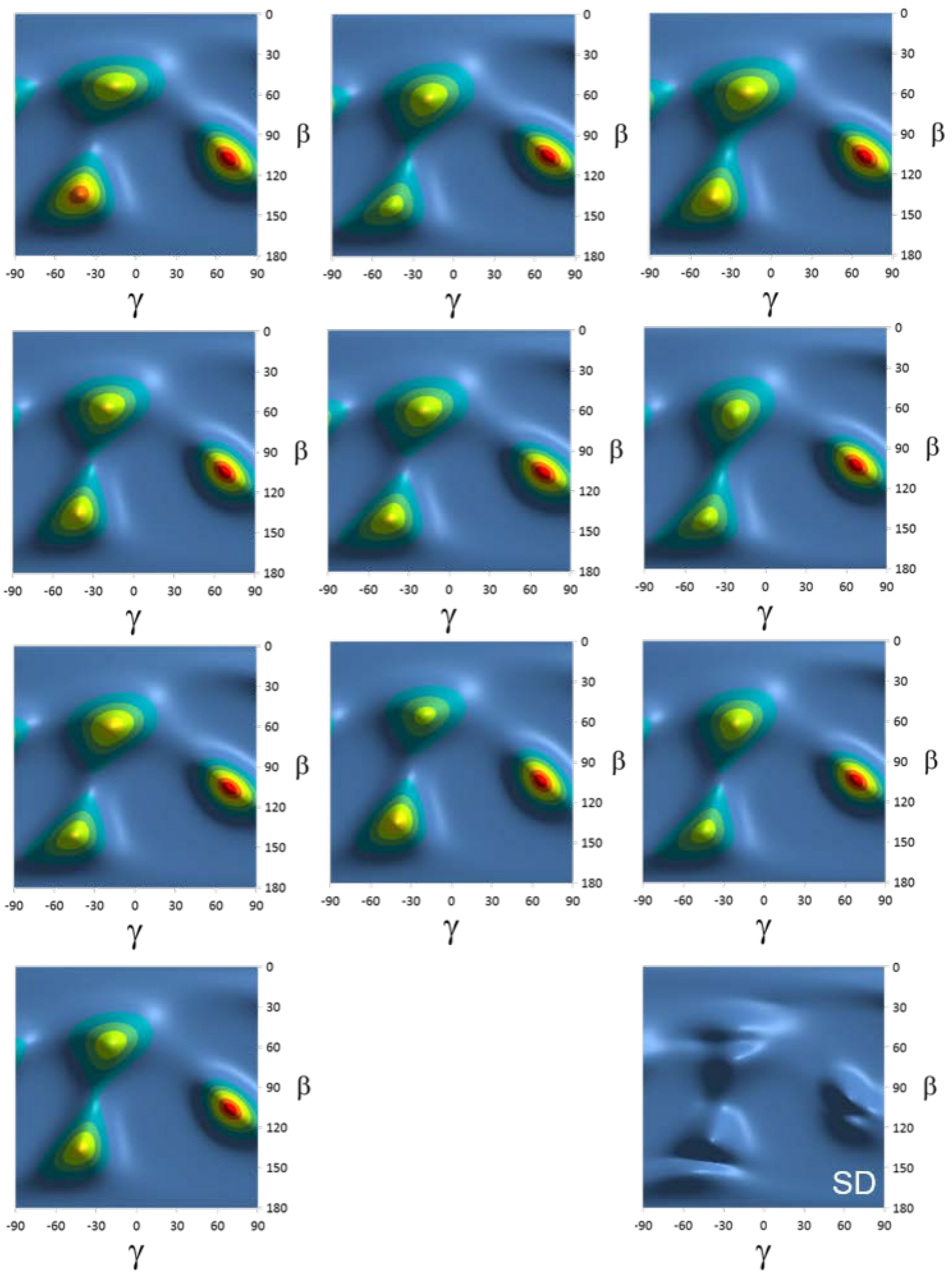

Figure S6. Reproducibility of maximum entropy orientation distributions of the RLC N-lobe ($(\beta,\gamma)_{DB}$) in relaxation, calculated using RLC coordinates from PDB entry 1SR6.

In order to assess the effects of measurement errors in the order parameters in **Table S2**, the mean values used for Fig. 3 in the main text were replaced by values chosen at random from normal distributions with the same mean and standard deviation. This procedure was repeated to give the ten contour maps shown to the top and left; the panel at the lower right, labeled SD, shows the standard deviation of these ten distributions.

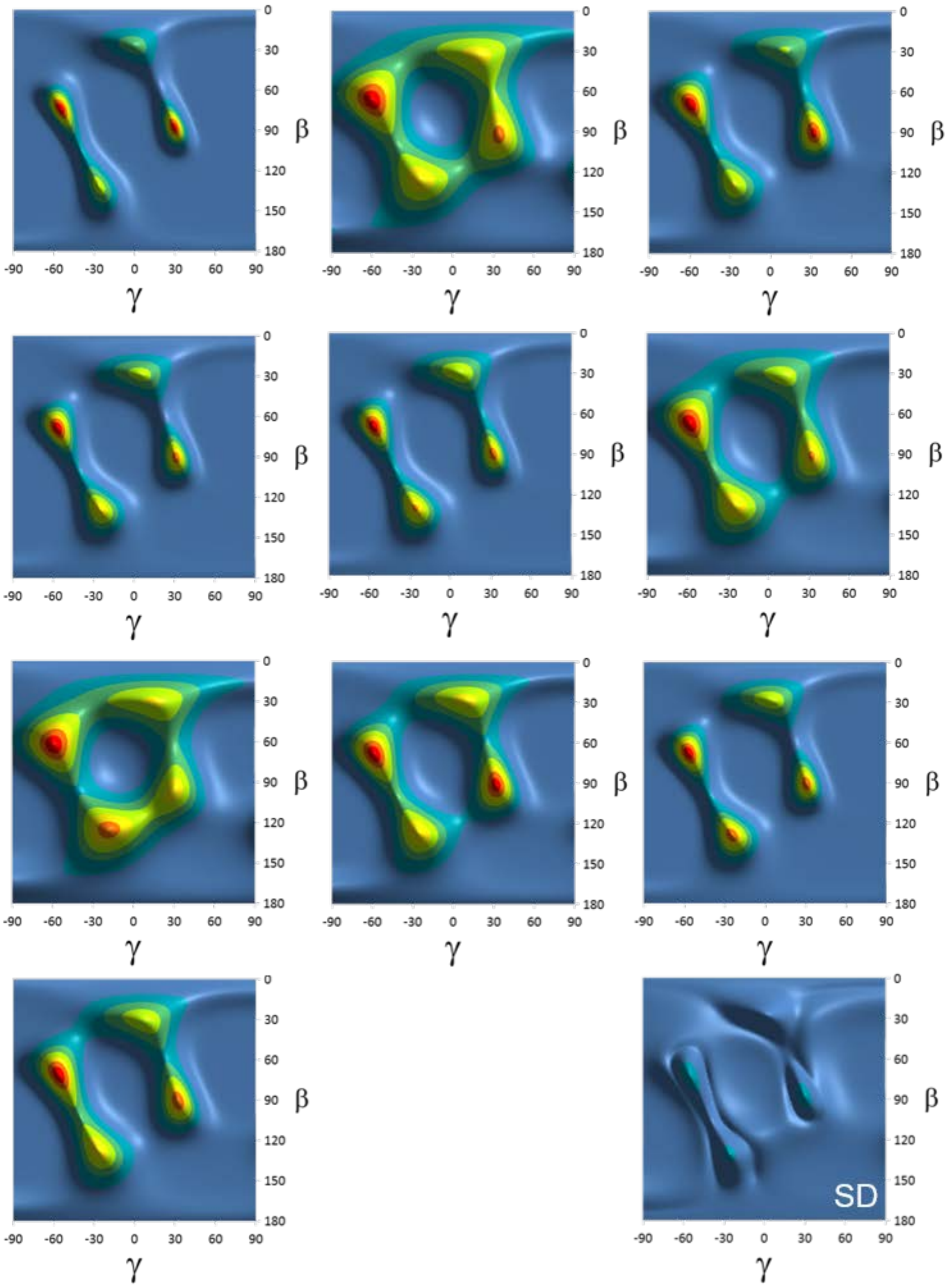

Figure S7. Reproducibility of maximum entropy orientation distributions of the RLC C-lobe ($(\beta,\gamma)_{EG}$) in relaxation, calculated using RLC coordinates from PDB entry 1SR6.

In order to assess the effects of measurement errors in the order parameters in **Table S2**, the mean values used for Fig. 4 in the main text were replaced by values chosen at random from normal distributions with the same mean and standard deviation. This procedure was repeated to give the ten contour maps shown to the top and left; the panel at the lower right, labeled SD, shows the standard deviation of these ten distributions.

Supplemental Tables

Table S1. Direct Comparison between EDTA- and CDTA exchange of BSR-cRLC-BC

		**CDTA Exchange**	**EDTA Exchange**
	Exchange Rate [%]	50 ± 5	12 ± 2
REL	$<P_{2d}>$	0.8063 ± 0.0143	0.8079 ± 0.0162
	$<P_2>$	-0.0009 ± 0.0078	-0.0106 ± 0.0067
	$<P_4>$	-0.0237 ± 0.0130	-0.0210 ± 0.0136
ACT	$<P_{2d}>$	0.8015 ± 0.0151	0.8027 ± 0.0110
	$<P_2>$	0.0853 ± 0.0050	0.0762 ± 0.0043
	$<P_4>$	-0.0212 ± 0.0150	-0.0175 ± 0.0085
	Force Before Exchange [kPa]	36.5 ± 3.5	37.6 ± 2.2
	Force After Exchange [kPa]	31.4 ± 2.7	33.6 ± 2.5
	Force Recovery [%]	87 ± 6 (n=7)	89 ± 3 (n=5)

Statistical significance of differences between values was assessed using the paired Student's t-test: †$P < 0.05$; ‡$P < 0.01$; *$P < 0.001$

Table S2. Order parameters for the N- and C-lobe RLC probes during relaxation, active isometric contraction and rigor.

Order parameters $<P_{2d}>$, $<P_2>$ and $<P_4>$ for the orientation of the BSR-cRLC probes during relaxation, active isometric contraction and rigor in permeabilized right ventricular trabeculae. Values indicate mean ± S.E.M. ($n = 5$). Statistical significance of differences between values was assessed using the paired Student's t-test: †$P < 0.05$; ‡$P < 0.01$; *$P < 0.001$

	Relaxed			Active			Rigor		
	$<P_{2d}>$	$<P_2>$	$<P_4>$	$<P_{2d}>$	$<P_2>$	$<P_4>$	$<P_{2d}>$	$<P_2>$	$<P_4>$
BSR-cRLC-A	0.7033 ± 0.0101	0.0011 ± 0.0074	0.1520 ± 0.0124	0.6922 ± 0.0185	0.0241 ± 0.0109†	0.1399 ± 0.0090	0.6774 ± 0.0177	0.1774 ± 0.0100*	0.1449 ± 0.0233
BSR-cRLC-BC	0.8165 ± 0.0112	-0.0046 ± 0.0057	-0.0404 ± 0.0066	0.8095 ± 0.0143	0.0592 ± 0.0074*	-0.0297 ± 0.0100†	0.8180 ± 0.0137	0.0386 ± 0.0139*	-0.0079 ± 0.0109*
BSR-cRLC-C	0.5985 ± 0.0094	-0.0636 ± 0.0084	0.1944 ± 0.0166	0.5773 ± 0.0079	-0.0637 ± 0.0099	0.2283 ± 0.0107*	0.5851 ± 0.0158	-0.1721 ± 0.0246	0.2501 ± 0.0313*
BSR-cRLC-D	0.6772 ± 0.0213	0.0087 ± 0.0077	0.0810 ± 0.0335	0.6615 ± 0.0257	-0.0198 ± 0.0043*	0.1382 ± 0.0376*	0.6769 ± 0.0191	-0.1008 ± 0.0105*	0.0889 ± 0.0226
BSR-cRLC-E	0.6424 ± 0.0153	0.1359 ± 0.0127	0.1259 ± 0.0175	0.6415 ± 0.0163	0.1003 ± 0.0138‡	0.1225 ± 0.0156	0.6637 ± 0.0166	-0.0508 ± 0.0163*	0.0407 ± 0.0169*
BSR-cRLC-F	0.8972 ± 0.0235	0.0275 ± 0.0028	-0.0426 ± 0.0196	0.8922 ± 0.0240	0.0117 ± 0.0068†	-0.0546 ± 0.0167	0.8948 ± 0.0217	-0.1025 ± 0.0153*	-0.0416 ± 0.0199
BSR-cRLC-G	0.6380 ± 0.0096	0.0155 ± 0.0057	-0.0171 ± 0.0222	0.6215 ± 0.0124	0.0220 ± 0.0074	0.0038 ± 0.0257†	0.6227 ± 0.0104	0.0072 ± 0.0093	0.0019 ± 0.0281
BSR-cRLC-FG	0.6541 ± 0.0084	0.0347 ± 0.0072	0.0982 ± 0.0110	0.6412 ± 0.0097	0.0262 ± 0.0051†	0.1172 ± 0.0085	0.6371 ± 0.0117	-0.0026 ± 0.0075*	0.0919 ± 0.0119

Table S3. Mean orientation θ_{ME} [°] of RLC probes with respect to the filament axis and standard deviation σ_{ME} of one - dimensional maximum entropy distributions calculated from the measured order parameters $\langle P_2 \rangle$ and $\langle P_4 \rangle$

	Relaxed		Active		Rigor	
	θ_{ME}	σ_{ME}	θ_{ME}	σ_{ME}	θ_{ME}	σ_{ME}
BSR-cRLC-A	57.3 ± 0.4	26.4 ± 0.3	56.2 ± 0.6	26.3 ± 0.3	48.6 ± 0.5*	26.7 ± 0.7
BSR-cRLC-BC	56.8 ± 0.8‡	20.1 ± 0.5	54.6 ± 0.2‡	20.7 ± 0.7	55.4 ± 0.1†	21.3 ± 0.7
BSR-cRLC-C	60.7 ± 0.4	26.8 ± 0.5	60.8 ± 0.5	27.7 ± 0.4	66.6 ± 1.4‡	25.8 ± 0.9
BSR-cRLC-D	56.9 ± 0.4	24.1 ± 0.8	58.4 ± 0.1‡	25.4 ± 1.0	62.1 ± 0.3*	21.2 ± 1.6*
BSR-cRLC-E	50.3 ± 0.8‡	26.3 ± 0.5	52.4 ± 0.7‡	26.6 ± 0.7	59.8 ± 0.9‡	22.2 ± 0.7*
BSR-cRLC-F	55.5 ± 0.2	20.7 ± 0.7	56.3 ± 0.3†	20.1 ± 0.6	61.8 ± 0.8†	18.3 ± 0.5*
BSR-cRLC-G	56.2 ± 0.3	21.4 ± 0.7	55.9 ± 0.4	22.2 ± 0.8	56.7 ± 0.5	21.9 ± 0.8
BSR-cRLC-FG	55.5 ± 0.4	24.0 ± 0.3	56.0 ± 0.3	25.7 ± 0.3	57.4 ± 0.4†	24.6 ± 0.3

Values indicate mean ± SEM (n=5). Statistical significance of differences between values was assessed using the paired student's t-test: †$P < 0.05$; ‡$P < 0.01$; *$P < 0.001$

Supporting References

1. Risal, D., S. Gourinath, D. M. Himmel, A. G. Szent-Gyorgyi, and C. Cohen. 2004. Myosin subfragment 1 structures reveal a partially bound nucleotide and a complex salt bridge that helps couple nucleotide and actin binding. *P Natl Acad Sci USA* 101:8930-8935.
2. Brown, J. H., V. S. Kumar, E. O'Neall-Hennessey, L. Reshetnikova, H. Robinson, M. Nguyen-McCarty, A. G. Szent-Gyorgyi, and C. Cohen. 2011. Visualizing key hinges and a potential major source of compliance in the lever arm of myosin. *P Natl Acad Sci USA* 108:114-119.
3. Rayment, I., W. R. Rypniewski, K. Schmidt-Base, R. Smith, D. R. Tomchick, M. M. Benning, D. A. Winkelmann, G. Wesenberg, and H. M. Holden. 1993. Three-dimensional structure of myosin subfragment-1: a molecular motor. *Science* 261:50-58.
4. Lawson, J. D., E. Pate, I. Rayment, and R. G. Yount. 2004. Molecular dynamics analysis of structural factors influencing back door pi release in myosin. *Biophys J* 86:3794-3803.
5. Kinoshita, E., E. Kinoshita-Kikuta, K. Takiyama, and T. Koike. 2006. Phosphate-binding tag, a new tool to visualize phosphorylated proteins. *Mol. Cell Proteomics* 5:749-757.
6. Gourinath, S., D. M. Himmel, J. H. Brown, L. Reshetnikova, A. G. Szent-Gyorgyi, and C. Cohen. 2003. Crystal structure of scallop Myosin s1 in the pre-power stroke state to 2.6 a resolution: flexibility and function in the head. *Structure* 11:1621-1627.
7. Debreczeni, J. E., L. Farkas, V. Harmat, C. Hetenyi, I. Hajdu, P. Zavodszky, K. Kohama, and L. Nyitray. 2005. Structural evidence for non-canonical binding of Ca2+ to a canonical EF-hand of a conventional myosin. *J Biol Chem* 280:41458-41464.
8. Romano, D., B. D. Brandmeier, Y. B. Sun, D. R. Trentham, and M. Irving. 2012. Orientation of the N-terminal lobe of the myosin regulatory light chain in skeletal muscle fibers. *Biophys J* 102:1418-1426.
9. Brack, A. S., B. D. Brandmeier, R. E. Ferguson, S. Criddle, R. E. Dale, and M. Irving. 2004. Bifunctional rhodamine probes of Myosin regulatory light chain orientation in relaxed skeletal muscle fibers. *Biophys J* 86:2329-2341.
